# A multicomponent reaction platform towards multimodal near-infrared BODIPY dyes for STED and fluorescence lifetime imaging†

**DOI:** 10.1039/d2cb00168c

**Published:** 2022-08-25

**Authors:** Antonio Fernandez, Nicola Kielland, Ashraff Makda, Neil O. Carragher, M. Carmen González-García, Laura Espinar-Barranco, Juan A. González-Vera, Angel Orte, Rodolfo Lavilla, Marc Vendrell

**Affiliations:** Centre for Inflammation Research, The University of Edinburgh Edinburgh UK marc.vendrell@ed.ac.uk; Dpt Organic Chemistry, Faculty of Chemistry, University of Murcia Spain ajfvargas@ugr.es; Laboratory of Medicinal Chemistry, Faculty of Pharmacy and Institute of Biomedicine (IBUB), University of Barcelona Spain; Institute of Genetics and Cancer, The University of Edinburgh Edinburgh UK; Nanoscopy-UGR Laboratory, Facultad de Farmacia, Universidad de Granada Granada Spain

## Abstract

We report a platform combining multicomponent reaction synthesis and automated cell-based screening to develop biocompatible NIR-BODIPY fluorophores. From a library of over 60 fluorophores, we optimised compound NIRBD-62c as a multimodal probe with suitable properties for STED super-resolution and fluorescence lifetime imaging. Furthermore, we employed NIRBD-62c for imaging trafficking inside cells and to examine how pharmacological inhibitors can alter the vesicular traffic between intracellular compartments and the plasma membrane.

## Abstract 2

By combining synthetic multicomponent reactions and cell-based screenings, we identified NIRBD-62c as a multimodal probe for STED and fluorescence lifetime imaging of the vesicular traffic between intracellular compartments and the plasma membrane.

## Graphical abstract

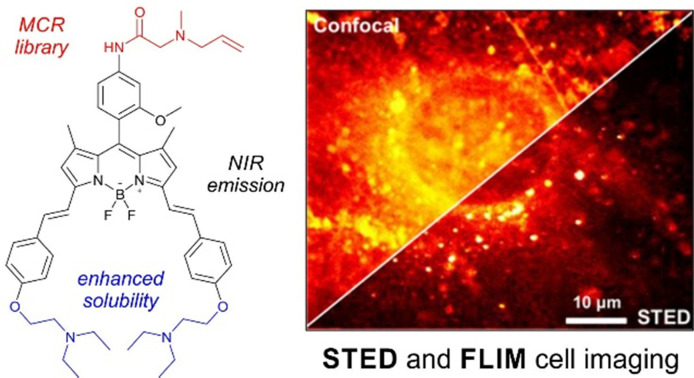

## Introduction

Intracellular trafficking involves the movement of biomolecules from synthetic compartments to locations where they can be released to the extracellular space.^1–4^ Cellular trafficking is heavily compartmentalized within a dynamic endomembrane network of subcellular organelles. The main vesicular organelles found in eukaryotic cells are lysosomes, the endoplasmic reticulum (ER) and the Golgi apparatus. The exchange of cargoes, membrane components and solutes between these organelles is a rapid process with an essential role in cellular homeostasis;^5,6^ therefore, disruptions in intracellular trafficking can lead to dysfunctional organelles and subsequent pathological disorders.^7,8^ As a result, chemical tools for real-time monitoring of vesicular transport inside live cells are crucial to understand their pathophysiology and to identify disease-specific signatures.^9–12^

Recent advances in optical imaging have opened new opportunities for the design of chemical tools to monitor intracellular trafficking.^13–19^ Among these, fluorescent probes are powerful tools for both sensing and optical imaging because of their high sensitivity, specificity and versatility.^20–24^ In addition, technical developments during the last decade have enabled the visualization of physiological processes with enhanced subcellular resolution.^25–27^ Different super-resolution microscopy modalities have been designed to overcome the conventional diffraction limit, with STimulated Emission Depletion (STED) being one of the most widespread techniques.^28–30^ Briefly, in STED a diffraction-limit region is excited at one wavelength while a super-imposed, high-power, red laser beam is projected to deplete most of the lateral emission and leave only a central focal spot with diameter under the diffraction limit.^31^ Using this approach, STED microscopy can provide fluorescence images with spatial resolution under 50 nm and temporal resolution in the range of milliseconds, offering a means to image the dynamics of intracellular functions in live cells.^32^

Near-infrared (NIR) fluorophores are excellent tools for tracking biological processes inside cells in a non-invasive manner.^33–39^ In addition to minimizing the impact of tissue background fluorescence and maximizing photon penetration, STED depletion is more efficient in fluorophores excited by red and NIR light sources; however, the number of synthetic NIR fluorophores for super-resolution imaging of subcellular organelles remains limited.^40–42^ Therefore, the development of organelle-targeted responsive NIR fluorophores is an important step in the design of new approaches to visualize dynamic processes associated with intracellular trafficking.

Among the different organic fluorescent structures, we focused this work on the 4,4-difluoro-4-bora-3a,4a-diaza-s-indacene (BODIPY) scaffold.^43–45^ BODIPY is a widely employed fluorophore in optical imaging because of its excellent photophysical properties and cell permeability, which favours rapid accessibility to the endomembrane network of intracellular compartments. Recently, our group has demonstrated the suitability of BODIPY-based fluorophores for imaging membrane-associated events (*e.g.*, apoptosis) and receptor-mediated endocytic transport.^15,20,46,47^ Furthermore, we have demonstrated that the BODIPY core can be chemically diversified using multicomponent reactions to produce libraries of activatable fluorophores.^48^ In view of this, we envisioned that synthetic methodologies based on multicomponent reactions would accelerate the synthesis and optimization of new probes for super-resolution microscopy in the NIR range. Herein, we present the chemical synthesis of a new collection of NIR BODIPY probes and their validation for imaging cellular trafficking using STED and fluorescence lifetime imaging.

## Results and discussion

### Design and synthesis of a library of pH-activatable NIR BODIPYs

**Fig. 1:**
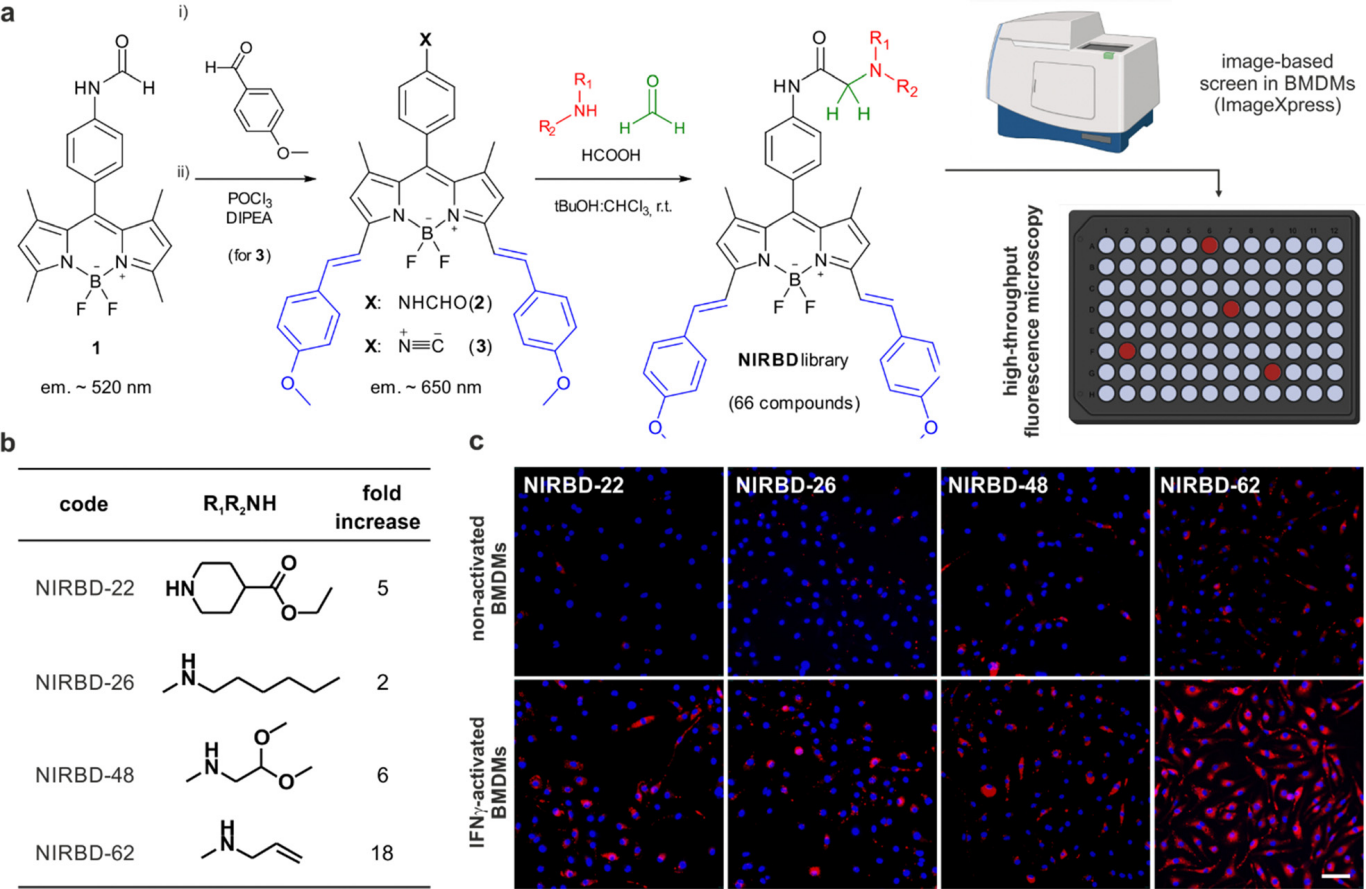
Synthesis and screening of the NIRBD library. (a) Chemical synthesis of NIRBD fluorophores and high-throughput screening in BMDMs. (b) Chemical structures and fluorescence fold increase in acid pH *vs.* neutral pH of selected NIRBD compounds. (c) Representative microscopy images of live BMDMs (20×) after incubation with Hoechst (blue) and NIRBD dyes (red). Scale bar: 50 μm.

Discovered by Lieke in 1859,^49^ isonitriles, which formally feature divalent carbon atoms, have become a pivotal functional group for multicomponent reaction chemistry and are broadly used in the modern production of diversity-oriented libraries of peptides and small molecules.^50–53^ Isonitrile-based multi-component reactions constitute a step forward in fluorophore derivatization;^54^ however, to the best of our knowledge, they have not been previously reported for the diversification of NIR BODIPY dyes. Based on our experience with pH-activatable fluorophores for monitoring vesicle trafficking, we designed a library of NIR BODIPY fluorophores with the formamide 2 (Fig. 1) as the main precursor, enabling *in situ* generation of isonitriles and subsequent derivatization into pH-sensitive fluorophores *via* multicomponent reaction chemistry. We synthesized the NIR-emitting formamide 2 by performing a double Knoevenagel condensation between *p*-anisaldehyde and the green-fluorescent BODIPY formamide 1 (Fig. 1). As expected, the condensation reaction took place between the α-methyl groups in the positions 3 and 5 of the BODIPY scaffold,^55,56^ yielding the double adduct in good yields (around 60%). The π-extended BODIPY formamide was dehydrated with POCl_3_ and DIPEA to generate the isonitrile 3 in quantitative yields, being the starting material to generate a collection of NIR BODIPY (NIRBD) dyes.

To study the versatility and application of the isonitrile 3 for the combinatorial generation of novel NIR fluorophores, we subjected the isonitrile 3 to the Ugi three-component reaction using formic acid, formaldehyde as a non-stereogenic carbonyl input and a range of over 60 structurally-diverse amines to generate the corresponding α-aminoamides (Fig. 1). Because α-aminoamides can affect the push-pull dipole in a pH-dependent manner, we envisaged that they would be suitable chemical groups to generate profluorophores that would turn-on in the acidic environments found in intracellular vesicles (*e.g.*, lysosomes, endosomes, phagosomes). Using this approach, we synthesized a library of 66 new NIR BODIPYs (Table S1, ESI† for detailed structures and characterization). Next, we performed automated high content image-based fluorescence screening in non-activated and phagocytic bone marrow-derived macrophages (BMDMs) to identify NIR fluorophores that would preferentially emit in phagocytic cells with enhanced trafficking activity. We ran a fluorescence-based screening where we cultured BMDMs in the absence or presence of interferon (IFN)-γ,^57^ followed by incubation with the NIRBD dyes. We acquired fluorescence microscopy images, using the ImageXpress high content screening platform, in both non-activated and IFNγ-activated cells and identified NIRBD dyes (NIRBD-22,26,48,62) with brighter emission in phagocytic cells (Fig. 1). Further *in vitro* assays at different pH values confirmed that all shortlisted NIRBD compounds were brighter in acidic environments when compared to the pH-insensitive isonitrile 3 (Fig. S1, ESI†).

After image analysis, we concluded that the *N*-allylmethylamine derivative NIRBD-62 showed the highest fold fluorescence increase between non-activated and activated BMDMs (Fig. 1), which was also replicated *in vitro* (Fig. S1, ESI†). Therefore, we selected this amine for further optimization of the NIRBD scaffold to generate new STED fluorophores for imaging intracellular trafficking in live cells.

### Optimization of water-soluble NIRBD fluorophores

**Fig. 2:**
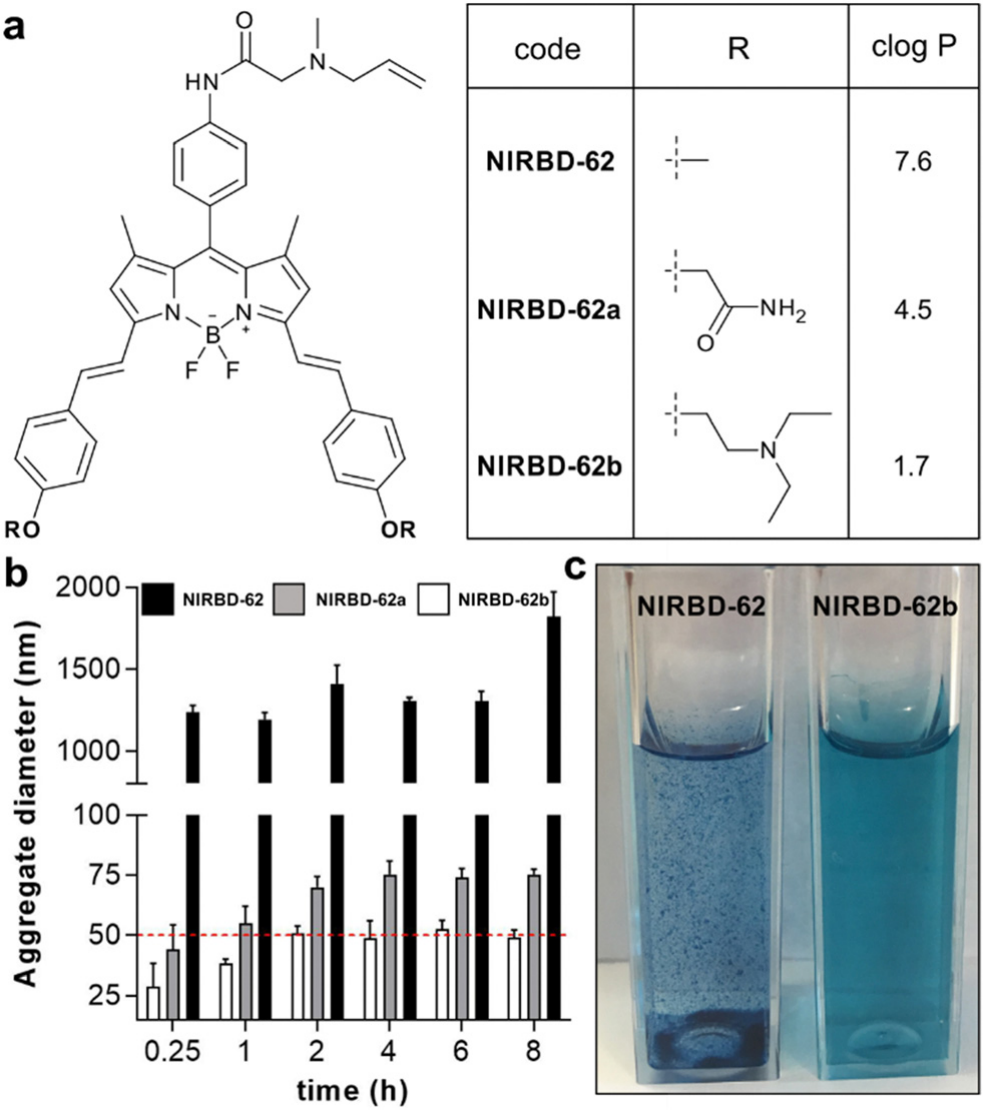
Optimization of the water solubility profile of NIR-BODIPY fluorophores. (a) Chemical structures of the styryl-functionalized NIRBD dyes and predicted *c* log *P* values for each derivative (chemical synthesis and full characterization in ESI†). (b) Time-course analysis of aggregate formation of NIRBD-62 analogues in aqueous media (100 μM in PBS) determined by dynamic light scattering at 25 °C for 8 h. Relative threshold for solubility at aggregates under 50 nm diameter (red). Values represented as means ± SD (*n* = 3). For compound NIRBD-62b, no detectable aggregates were found at lower concentrations (1–5 μM in PBS). (c) Representative pictograms of cuvettes containing solutions of compounds NIRBD-62 and NIRBD-62b in aqueous media (100 μM in PBS).

One of the most common limitations of NIR fluorophores is their reduced compatibility in aqueous media, which can lead to large aggregates and imaging artifacts. We analyzed the water solubility properties of NIRBD-62 and observed significant aggregation in phosphate buffer saline (PBS, Fig. 2), which hampered its utility in live-cell imaging experiments. NIR fluorophores, including BODIPYs and cyanines, can be readily modified with water-solubilizing groups (*e.g.*, sulfonates);^58^ however, the inclusion of negative charges within the NIRBD core might impair its affinity for the lipophilic domains of the endomembrane network. Therefore, we decided to optimize the chemical structure of NIRBD-62 by fine-tuning the styryl fragments of the BODIPY core (Fig. 2).

Based on predicted log *P* values, we selected two commercially available aldehydes that could decrease the lipophilicity of NIR BODIPYs to prepare more water-soluble analogues of NIRBD-62 (NIRBD-62a and NIRBD-62b, Fig. 2 and Fig. S2, ESI†). We synthesized NIRBD-62a and NIRBD-62b following the same strategy outlined in Fig. 1 (for full synthetic and characterization details, see ESI†). First, we performed Knoevenagel-like condensations using the two new aldehydes and the BODIPY formamide 1. The two NIR-BODIPY formamides were converted to the respective isonitriles and derivatized with *N*-allylmethylamine in a multicomponent approach with formaldehyde and formic acid to render the final products in moderate overall yields (around 20%) and high purities (>95%). Next, we compared the water solubility of the original compound NIRBD-62 and the new analogues by measuring the formation of aggregates over time using dynamic light scattering. We observed that both compounds NIRBD-62a and NIRBD-62b showed a markedly reduced tendency to form aggregates in PBS when compared to NIRBD-62 (Fig. 2), with NIRBD-62b being the most water-soluble analogue. Furthermore, the qualitative analysis of 100 μM NIRBD solutions in PBS showed much reduced aggregation in the case of NIRBD-62b, even after the compound had been in aqueous solution for several hours, which features its good water solubility. Finally, we confirmed that the modification of the styryl group in NIRBD-62b did not alter the ability to label intracellular compartments in phagocytic cells (Fig. S3 and S4, ESI†).

### STED and fluorescence lifetime imaging using NIR-BODIPYs

**Fig. 3:**
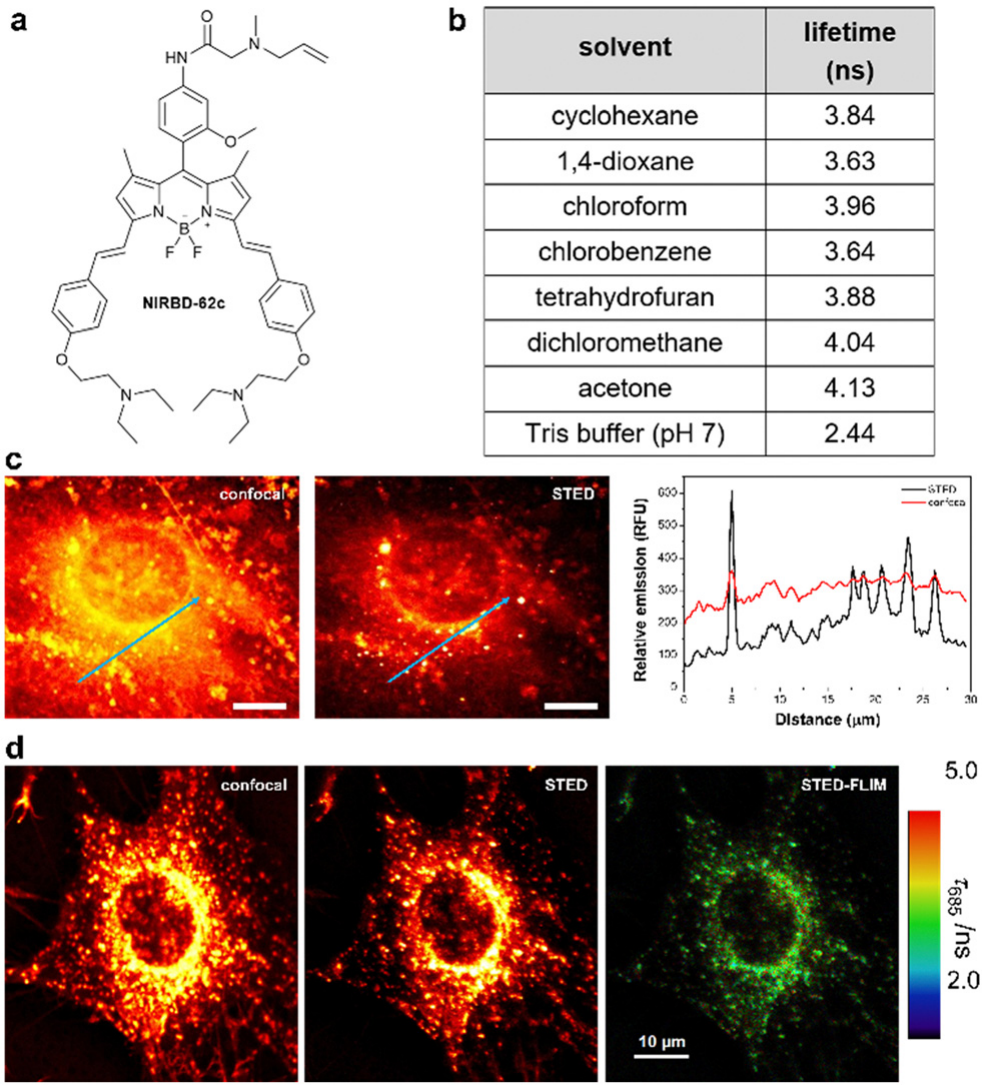
NIRBD-62c is a suitable fluorophore for multimodal STED and fluorescence lifetime imaging. (a) Chemical structure of the fluorophore NIRBD-62c (chemical synthesis and full characterization in ESI†). (b) Fluorescence lifetimes (*τ*) of compound NIRBD-62c in different solvents determined by time-resolved fluorimetry in single photon timing mode, using a 635 nm excitation laser. Values obtained from global fits of 3 measurements at different emission wavelengths, showing associated errors in the fittings lower than 0.03 ns in all cases. (c) Representative fluorescence microscopy images and associated intensity plot profiles of MC3T3-E1 osteoblasts upon incubation with NIRBD-62c (100 nM) under conventional fluorescence confocal microscopy and STED microscopy. Both images were simultaneously acquired using one pulse of 640 nm excitation laser (for confocal) and three pulses of excitation + depletion laser (for STED). Emission was collected using a 685/70 bandpass filter. (d) Representative STED-FLIM pseudocolored image, using the same configuration as in (c) and after performing a pixelwise tail-fit of the fluorescence decay to estimate *τ* values.

STED microscopy is an excellent technique for high-resolution structural imaging^59^ but, unlike ratiometric imaging or fluorescence lifetime imaging (FLIM), it cannot provide quantitative information. FLIM uses the luminescence decay rate and determines the corresponding luminescence lifetime (*τ*) of a given fluorophore as the parameter of interest. Unlike intensity-based measurements, FLIM is concentration-independent and therefore avoids the need for normalization factors.^59,60^ Therefore, the recent integration of FLIM with super-resolution microscopy (*e.g.*, STED-FLIM),^61^ combines the advantages of both modalities. To assess the usefulness of the NIRBD dyes in advanced STED-FLIM experiments, we first determined the *τ* values of compound NIRBD-62b in different solvents and observed very low *τ* values (<2 ns), which hampered its utility for FLIM experiments. To address this point, we decided to include a methoxy group in the meso aryl ring of the NIR BODIPY scaffold that would minimize non-radiative decay *via* restriction of rotational processes.^62^ The resulting NIRBD-62c (Fig. 3 and ESI,† for chemical synthesis and full characterization) displayed very high fluorescence quantum yields (from 25% in water to >95% in 1,4-dioxane, Fig. S5, ESI†) and consistently high *τ* values around 4 ns in different environments, which would be readily distinguishable from the cellular background (*e.g.*, NADPH, flavonoids) in FLIM experiments (Fig. 3). Furthermore, we acquired the fluorescence spectra of NIRBD-62c in multiple solvents (Fig. S6, ESI†). Notably, in addition to showing polarity-dependent emission maxima around 645 ± 10 nm, the compound NIRBD-62c also exhibited secondary, high-energy maxima at 590 nm and additional longer emission bands ∼720 nm (Fig. S6, ESI†). This unique multiband emission profile makes NIRBD-62c an optimal NIR fluorophore for ratiometric fluorescence imaging.

Given the photophysical features of NIRBD-62c as a bright and water-compatible NIR-emitting fluorophore, we tested its capabilities for super-resolution STED and STED-FLIM microscopy. An important feature in organic fluorophores for STED microscopy is their photostability upon excitation with the high-power depletion laser. First, we examined NIRBD-62c and observed bright and consistent fluorescence emission >650 nm during the 60 μs per pixel irradiation regime typically used for STED microscopy. Next, we compared the resolution of fluorescence microscopy images of live mouse osteoblasts (MC3T3-E1) obtained sequentially under regular confocal microscopy and STED microscopy. As shown in Fig. 3, we observed a significant improvement in image resolution for the latter, which highlights the compatibility of compound NIRBD-62c for super-resolution imaging. Notably, STED images exhibited well-defined subcellular vesicles containing the fluorophore in the perinuclear region and close to the cellular membrane (Fig. 3).

**Fig. 4:**
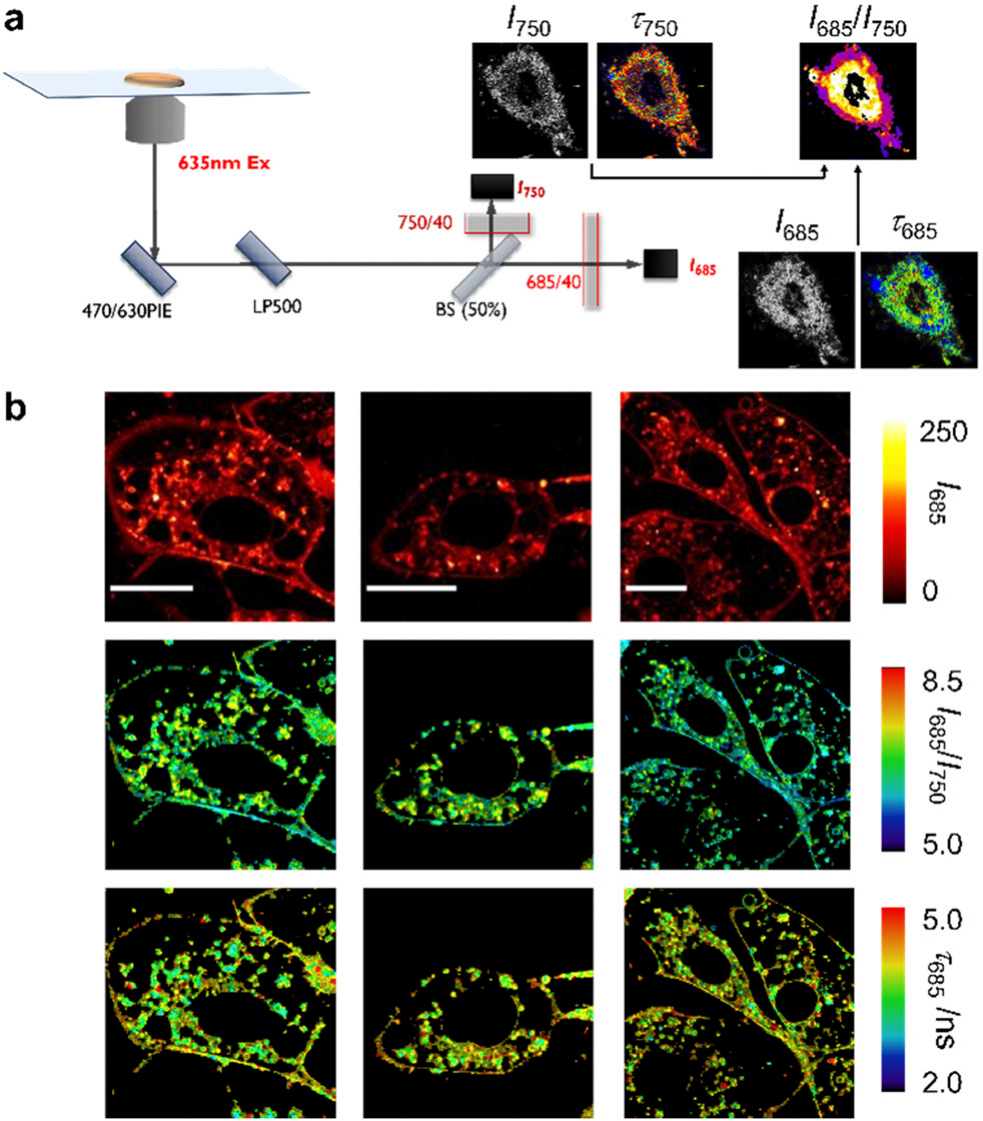
Multiparametric fluorescence microscope images of live mouse osteoblasts upon incubation with the compound NIRBD-62c. (a) Schematic illustration of the multiparametric fluorescence microscopy set-up. (b) Representative pseudocolored images from 3 different groups of MC3T3-E1 cells using the fluorescence intensity in the 685 nm channel (top row), the fluorescence ratiometric *I*_685_/*I*_740_ measurements (middle row) and the fluorescence lifetime in the 685 nm channel (bottom row). The cells were incubated with 100 nM of compound NIRBD-62c before imaging. Scale bar: 20 μm.

Next, we decided to exploit the multiband emission profile of NIRBD-62c to acquire ratiometric microscopy images in combination with FLIM in live osteoblasts. To perform these multiparametric imaging experiments, we used a dual-color FLIM set-up (Fig. 4), which consisted of a pulsed 635 nm excitation laser and two single-photon detection channels with bandpass filters of 685/70 (*I*_685_) and 750/40 (*I*_750_), allowing the reconstruction of both fluorescence lifetime and ratiometric images (*I*_685_/*I*_750_). The ratiometric images obtained from the polarity-sensitive fluorescence band >650 nm (close the NIRBD-62c maxima) and the long-emission band >700 nm (Fig. S6, ESI†) highlighted the sensitivity of compound NIRBD-62c to different subcellular microenvironments, with the brightest regions being around perinuclear compartments and slightly weaker staining detected on the cell membranes. Notably, such differences were detected under FLIM too. In this case, NIRBD-62c showed *τ* values around 4.2 ns (28 regions of interest, ROI) in the perinuclear regions whereas slightly longer *τ* values 4.4 ns (40 ROIs) were found in the cytoplasmic membrane (Fig. 4 and Movies S1 and S2, ESI†). Altogether, these observations highlight that compound NIRBD-62c can distribute across different compartments of the endomembrane network, and that its localization can be monitored by ratiometric fluorescence imaging as well as FLIM.

In order to examine the underlying mechanisms behind the environmental sensitivity of compound NIRBD-62c, we applied the methodology developed by Catalán to study its solvatochromic features.^63^ This methodology allows to correlate photophysical properties of a given fluorophore to the main features of different organic solvents, such as polarity, polarizability, acidity, and basicity. Specifically, we examined the absorption and emission maxima wavelengths of NIRBD-62c in multiple organic solvents (Fig. S6, ESI†) and found that the main solvent property that affected both absorption and emission maxima was polarizability (Fig. S7, ESI†). This behavior is likely due to the large size of NIRBD-62c, which leads to the dispersion interactions between the electron clouds of the fluorophore and the solvents defining ground and excited-state stabilization upon solvation. In addition to polarizability, we also observed that the acidity and basicity of the media also contributed to the excited-state stabilization. This is expected for pH-sensitive fluorophores like the NIRBD compounds and suggests that hydrogen bonding interactions are involved in the relaxation processes. Moreover, we analyzed the *I*_685_/*I*_750_ ratios of compound NIRBD-62c*vs.* the polarity and polarizability of solvents, and observed a direct correlation between the intensity ratios and both parameters, with the *I*_685_/*I*_750_ ratio increasing with solvent polarity and decreasing with solvent polarizability. These results confirm that the *I*_685_/*I*_750_ ratios acquired in microscopy experiments can be considered as direct reporters of the fluorophore's microenvironment.

**Fig. 5:**
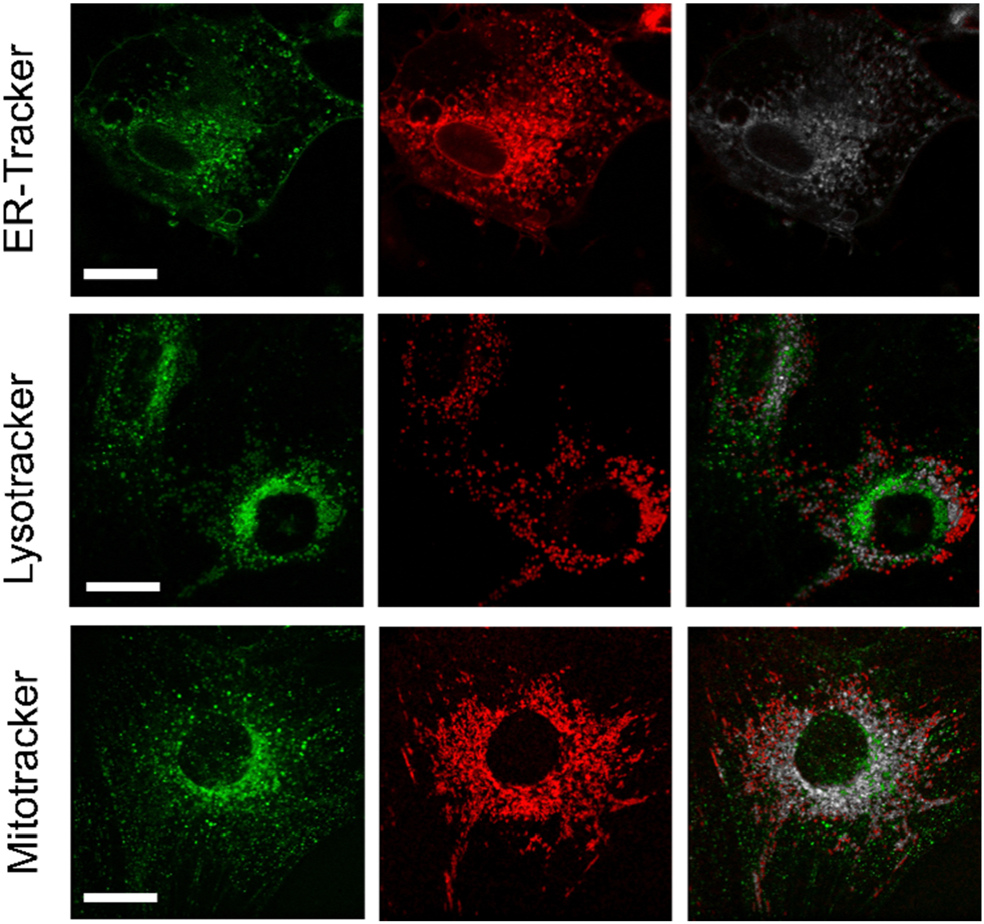
Co-localization fluorescence microscopy of compound NIRBD-62c with organelle fluorescent trackers. Representative confocal microscopy images from live-cell osteoblasts upon incubation with compound NIRBD-62c (green) and commercially available organelle trackers (red). Co-localization areas are highlighted in grey in the merged fluorescence images. Scale bar: 20 μm.

Next, we decided to examine the distribution of compound NIRBD-62c in different subcellular organelles. For these experiments, we performed co-localization experiments where we incubated cells with both NIRBD-62c and different commercially available trackers (*i.e.*, ER-Tracker, Lysotracker and Mitotracker). As shown in Fig. 5, most of the intracellular staining of compound NIRBD-62c was found in vesicular structures around the ER, with little accumulation in mitochondria or lysosomes. This result, together with the partial localization of the fluorophore observed in the cellular membrane (Fig. 4), suggest that compound NIRBD-62c holds potential as a fluorophore to monitor intracellular trafficking processes from the ER to the plasma membrane and the extracellular space.

Finally, we decided to study whether the fluorescence readouts of compound NIRBD-62c could be used to monitor alterations in intracellular trafficking. For these experiments, we used some reported pharmacological inhibitors; specifically: (1) dynasore, a non-competitive dynamin inhibitor that blocks endocytic pathways^64^ and (2) brefeldin A (BFA),^65^ an antiviral drug that inhibits protein transport from the ER to the Golgi complex.

**Fig. 6:**
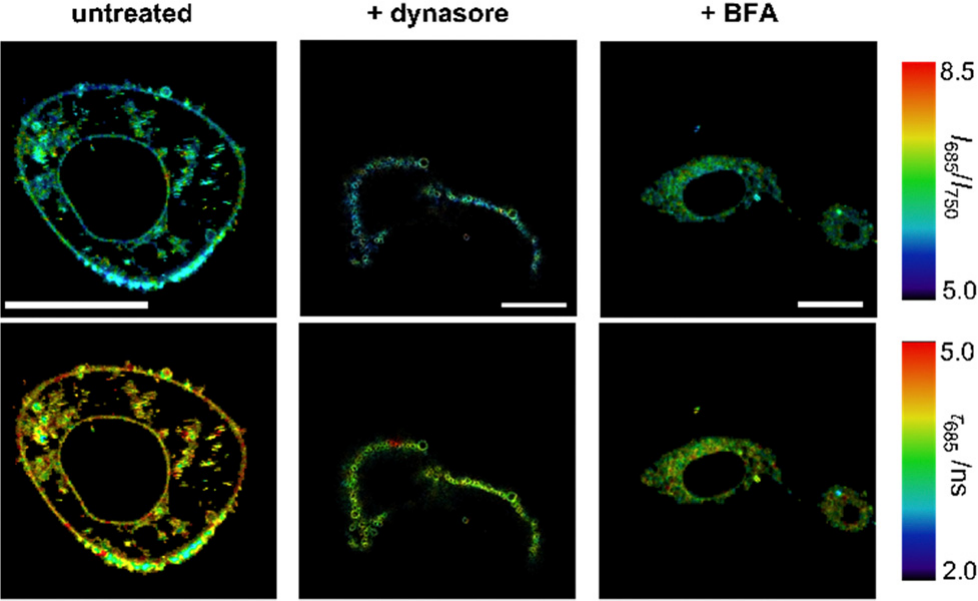
Multiparametric fluorescence microscopy of NIRBD-62c in cells upon treatment with different pharmacological inhibitors. Representative pseudocolored images from 3 different groups of cells (all incubated with NIRBD-62c, 100 nM) that were untreated, treated with dynasore (80 μM) or BFA (5 μM). Microscopy images display ratiometric measurements (top row) as well as fluorescence lifetime measurements (bottom row). For ratiometric images, pseudocolors indicate the fluorescence intensity ratios of compound NIRBD-62c at 685 nm *vs.* 750 nm. For FLIM images, pseudocolors indicate the variations in the fluorescence lifetimes (emission: 685 nm) of NIRBD-62c in different subcellular regions. Scale bar: 20 μm.

First, we observed that the fluorescence emission of compound NIRBD-62c was drastically reduced (*i.e.*, >5-fold decrease) upon treatment with dynasore when compared to untreated or BFA-treated cells (Fig. S8, ESI†). Moreover, dynasore-treated cells exhibited the compound mostly trapped in O-shape invaginations near the plasma membrane (Fig. 6), consistent with the effect of dynasore as a dynamin inhibitor, which leads to clathrin-coated vesicles trapped in the plasma membrane.^66^ This observation suggested that endocytosis plays an important role in the internalization of NIRBD-62c. To further analyze this point, we incubated cells at 4 °C to minimize endocytic trafficking. In these experiments, we found bright staining in the ER-Golgi system but a drastically reduced number of intracellular vesicles (Fig. S9, ESI†), suggesting that the uptake of NIRBD-62c can proceed through both endocytic transport and passive diffusion. On the other hand, the treatment with BFA led to minor differences in fluorescence intensity (Fig. S8, ESI†) but drastic variations in the localization of compound NIRBD-62c. Specifically, the labelling of the plasma membrane was reduced in BFA-treated cells and led to the accumulation of the fluorophore in the perinuclear region. This observation suggests that the blockade of the transport between the ER and Golgi apparatus is important for vesicular trafficking to the cell membrane and subsequent exocytosis. Notably, in addition to the differences in intracellular localization, we corroborated this observation by FLIM. As shown in Fig. 6 and Fig. S8 (ESI†), NIRBD-62c displayed longer *τ* values in untreated cells with brighter plasma membrane localization than in BFA-treated cells – with brighter accumulation in perinuclear compartments, such as the ER-, which is in line with the fluorescence lifetimes found for compound NIRBD-62c in different subcellular regions.

## Conclusions

In summary, we have designed a platform for the synthesis and high-throughput screening of new NIR-BODIPY fluorophores that enable live-cell imaging of intracellular trafficking. Herein we report one of the first examples of isonitrile-functionalized NIR scaffolds and their derivatization by multicomponent reaction chemistry to generate collections of new fluorophores.

We envision that the adaptation of this synthetic strategy to other fluorophores will accelerate the design of NIR probes. From an initial library of 60+ NIR fluorophores, we have optimized NIRBD-62c as a single multimodal probe with good water solubility and suitable properties for ratiometric confocal imaging, STED super-resolution imaging and FLIM. We have applied multiparametric fluorescence imaging and pharmacological inhibitors to confirm that NIRBD-62c predominantly locates in the ER of live cells and can monitor intracellular traffic to the plasma membrane. NIRBD-62c and future derivatives hold potential as novel non-invasive imaging tools to study how molecules translocate across subcellular compartments.

## Author contributions

Conceptualization: A. F., N. K., R. L. and M. V.; funding acquisition: A. F., J. A. G. V., A. O., R. L., M. V.; investigation: A. F., N. K., A. M., M. C. G. G., L. E. B., J. A. G. V. and M. V.; resources: N. O. C.; supervision: N. O. C., J. A. G. V., A. O., R. L., M. V.; writing – original draft: A. F. and M. V.; writing – review & editing: A. F., N. O. C., A. O., R. L. and M. V.

## Conflicts of interest

The authors have no conflicts to declare.

## Supplementary Material

CB-003-D2CB00168C-s001

CB-003-D2CB00168C-s002

CB-003-D2CB00168C-s003
