# Supplementary material for: A multicomponent reaction platform towards multimodal near-infrared BODIPY dyes for STED and fluorescence lifetime imaging
Source: RSC Chem Biol. 2022 Aug 25;3(10):1251–9. doi: 10.1039/d2cb00168c (PMC9533399; doi:10.1039/d2cb00168c)
Supplement: CB-003-D2CB00168C-s003 [file CB-003-D2CB00168C-s003.pdf]

Electronic Supplementary Material (ESI) for RSC Chemical Biology.
This journal is © The Royal Society of Chemistry 2022

Electronic Supplementary Information

A Multicomponent Reaction Platform towards Multimodal Near-Infrared BODIPY Dyes for STED and Fluorescence Lifetime Imaging

Table of Contents

Materials and methods

Chemical synthesis and characterization

Spectroscopy and imaging protocols

Supplementary Figures

Supplementary Movies

Supplementary References

Materials and methods

Commercially available reagents were used without further purification. Thin-layer chromatography was conducted on Merck silica gel 60 F254 sheets and visualized by UV (254 and 365 nm). Silica gel (particle size 35–70 μm) was used for column chromatography. ^{1}H and ^{13}C spectra were recorded in a Bruker Avance 500 spectrometer (at 500 and 125 MHz, respectively). Data for ^{1}H NMR spectra are reported as chemical shift δ (ppm), multiplicity, coupling constant (Hz) and integration. Data for ^{13}C NMR spectra reported as chemical shifts relative to the solvent peak. HPLC-MS analysis was performed on a Waters Alliance 2695 separation module connected to a photodiode array detector and a ZQ Micromass mass spectrometer (ESI-MS) with a Phenomenex® column (C_{18}, 5 μm, 4.6×150 mm^2).

Chemical synthesis and characterization

Synthesis of NIRBD formamide **2** and isonitrile **3**

In a 50 mL round bottom flask with a Dean-Stark apparatus 4,4-difluoro-8-(4-formamidophenyl)-1,3,5,7-tetramethyl-4-bora-3a,4a-diaza-s-indacene **1**[1] (50 mg, 0.136 mmol), 4-methoxybenzaldehyde (74 mg, 0.544 mmol), *p*-toluenesulfonic acid (10 mg, 0.06 mmol), toluene (20 mL) and piperidine (0.6 mL) were added. After heating to reflux, the progress of the reaction was monitored by TLC and the reaction was stopped until the starting material disappeared completely. Then, the solvent was removed under reduced pressure and the residue was purified by column chromatography (CH_2Cl_2:MeOH, 9:1) to afford BODIPY formamide **2** (52 mg, 63% yield).

^{1}H NMR (400 MHz, DMSO) δ 10.47–10.34 (m, 1H), 8.40 – 8.31 (m, J = 1.8 Hz, 1H), 7.80 (d, J = 8.5 Hz, 2H), 7.58 (d, J = 8.8 Hz, 4H), 7.53 (d, J = 16.3 Hz, 2H), 7.41 (d, J = 11.7 Hz, 2H), 7.37 (d, J = 8.6 Hz, 2H), 7.04 (d, J = 8.8 Hz, 4H), 6.94 (s, 2H), 3.82 (s, 6H), 1.47 (s, 6H).

^{13}C NMR (100 MHz, DMSO) δ 160.4, 159.9, 152.1, 141.5, 139.0, 137.9, 136.6, 132.7, 129.2, 129.1, 128.8, 128.8, 119.4, 118.1, 115.9, 114.7, 55.3.

^{19}F NMR (376 MHz, DMSO) δ -136.1 (dd, J = 62.3, 22.6 Hz).

HRMS (m/z): (M+H$^+$) calcd. for $C_{36}H_{33}BF_2N_3O_3$: 604.2578; found 604.2573.

Afterwards, to a solution of BODIPY formamide **2** (50 mg, 83 mmol) in CH_2Cl_2 (10 mL), triethylamine (92 μL, 0,66 mmol) was added. The resulting mixture was cooled down at 0 ^{o}C and $POCl_3$ (19 μL, 0,21 mmol) was added slowly and the mixture was

stirred at 0 ^{o}C for 2 h. Then, a 2 M $NaHCO_3$ aqueous solution (10 mL) was added. The aqueous layer was extracted with CH_2Cl_2 (3 × 3 mL). The organic extracts were dried over $MgSO_4$, filtered off and evaporated under reduced pressure to yield BODIPY isonitrile **3** (99% conversion by HPLC-MS), which was used without further purification.

Synthesis of NIRBD library

For each amine (batches of 24 amines at a time), tBuOH (35 µL), BODIPY isonitrile **3** (10 µL of a 3 µM stock solution in $CHCl_3$), formaldehyde (10 µL of a 3 µM solution in $CHCl_3$), formic acid (10 µL of a 3 µM solution in $CHCl_3$) and the corresponding amine (10 µL of a 3 µM solution in $CHCl_3$) were mixed in a 2 mL eppendorf tube. The tubes were heated at 40 ^{o}C and stirred during 24 h, then opened up until complete evaporation of the solvent. The mixtures were analyzed by HPLC, and the reactions showing more than 80% conversion were selected. For each selected reaction, the mixture was re-dissolved in CH_2Cl_2 (50 µL) and purified by preparative TLC using hexane:EtOAc:MeOH mixtures. Final conversion and purity were estimated by HPLC-MS.

Table S1. Library of NIRBD probes and chemical characterization.

NIRBD code	Amine structure	$[M+H]_{calc}$	$[M+H]_{exp}$	tR (min)	HPLC purity 280 nm (%)
**1**	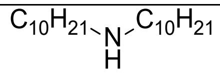	913.9	913.2	8.8	95
**2**	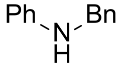	799.8	799.0	8.2	97
**3**	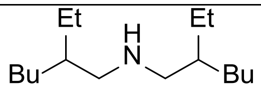	857.8	857.1	9.5	95
**4**	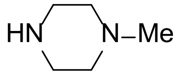	716.7	716.0	6.0	98
**5**	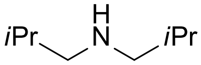	745.7	745.0	7.0	99
**6**	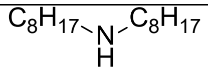	857.8	857.1	7.6	99
**7**	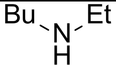	717.7	717.0	6.3	99
**8**	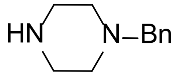	792.7	792.2	6.2	99
**9**	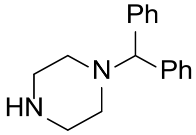	868.9	868.0	6.6	98
**10**	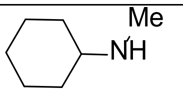	729.7	729.0	6.2	99
**11**	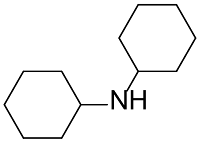	797.7	797.1	6.6	96
**12**	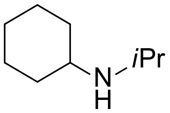	757.7	757.1	6.4	80

**13**	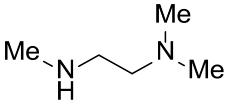	718.7	718.0	6.1	98
**14**	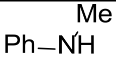	723.6	723.0	8.0	99
**15**	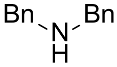	813.8	813.0	7.9	94
**16**	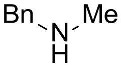	737.7	737.0	6.3	98
**17**	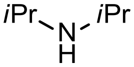	717.7	717.0	6.2	98
**18**	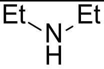	689.6	689.0	6.1	99
**19**	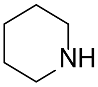	701.6	701.0	6.1	99
**20**	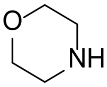	703.6	702.7	6.1	99
**21**	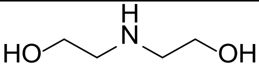	721.6	n.d.	5.9	98
**22**	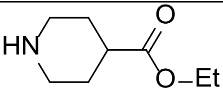	773.7	772.9	6.2	99
**23**	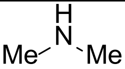	661.6	661.0	5.9	98
**24**	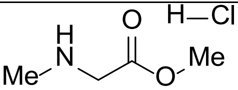	719.6	719.0	7.0	80
**25**	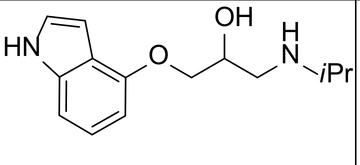	864.7	864.0	6.3	98
**26**	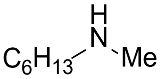	731.7	731.1	6.4	99

**27**	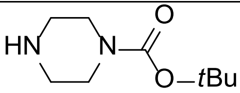	802.8	802.0	6.7	95
**28**	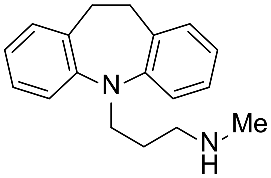	882.7	882.0	6.8	98
**29**	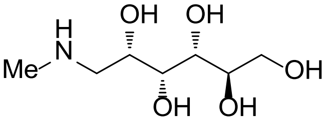	811.7	811.0	5.1	98
**30**	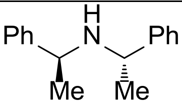	841.7	841.0	8.3	80
**31**	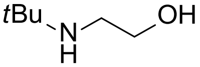	733.7	687.0	7.2	80
**32**	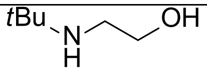	733.7	687.0	8.1	80
**33**	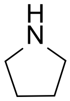	687.6	687.0	6.0	98
**34**	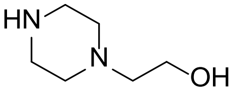	746.7	746.0	5.9	95
**35**	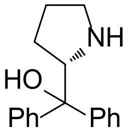	869.6	850.9	8.1	76
**36**	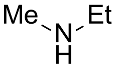	675.6	675.0	5.9	98
**37**	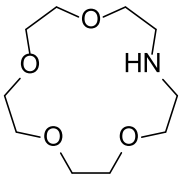	835.7	834.9	6.2	95

**38**	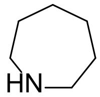	715.7	715.0	6.1	98
**39**	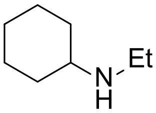	743.7	743.0	6.3	99
**40**	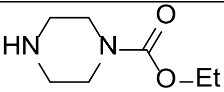	774.7	774.0	6.5	90
**41**	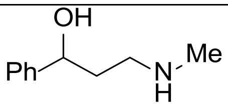	781.7	780.9	6.2	95
**42**	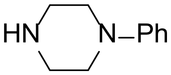	778.7	778.0	6.5	98
**43**	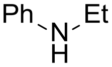	737.6	737.0	8.1	99
**44**	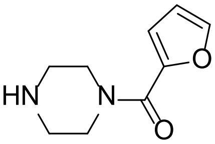	796.6	795.9	6.6	81
**45**	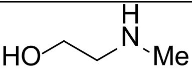	691.6	691.0	5.9	95
**46**	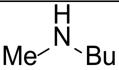	703.7	702.0	6.2	99
**47**	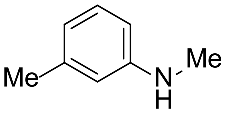	737.6	737.0	8.1	97
**48**	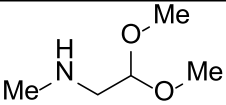	735.6	735.0	6.3	98
**49**	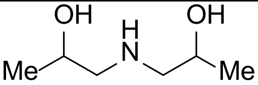	749.7	n.d.	6.0	90
**50**	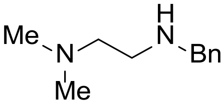	794.7	794.0	6.5	98

**51**	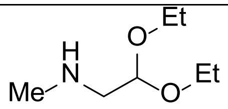	763.7	763.0	6.5	98
**52**	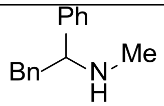	827.7	827.0	7.3	85
**53**	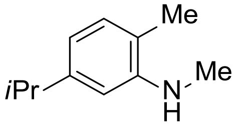	779.7	779.0	8.6	90
**54**	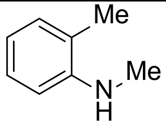	737.6	736.9	8.2	90
**55**	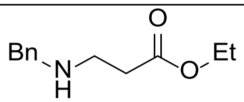	823.7	823.0	7.8	90
**56**	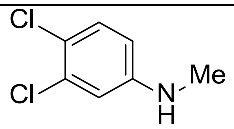	791.6	792.2	8.2	98
**57**	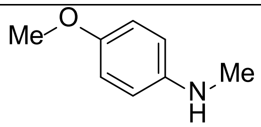	753.6	753.0	7.9	98
**58**	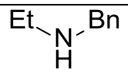	751.7	751.0	6.4	93
**59**	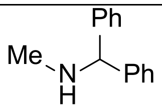	813.7	813.0	8.1	98
**60**	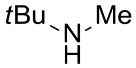	703.7	702.7	6.1	98
**61**	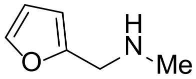	727.6	727.0	6.3	93
**62**	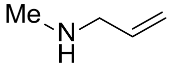	687.6	687.0	6.1	95

**63**	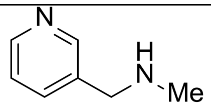	738.6	738.0	6.1	96
**64**	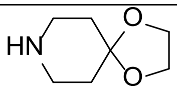	759.6	759.0	6.1	99
**65**	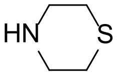	719.6	718.9	6.3	97
**66**	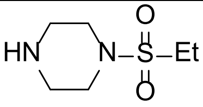	794.6	793.9	6.8	95

Synthesis of **NIRBD-62**

To a solution of *N*-(4-(5,5-difluoro-3,7-bis((E)-4-methoxystyryl)-1,9-dimethyl-5H-4l4,5l4-dipyrrolo[1,2-c:2',1'-f][1,3,2]diazaborinin-10-yl)phenyl)formamide (**2**) (40 mg, 0.066 mmol) in 6 mL $CHCl_3$ under inert atmosphere, 83 µL (0.462 mmol) DIPEA was added. The resulting mixture was cooled down at 0˚C and $POCl_3$ (15 µL, 0.198 mmol) was added dropwise. The reaction was stirred in the cold for 3 h. Then, 10 mL of 2 M $NaHCO_3$ were added, and the mixture was stirred for 5 min. The aqueous layer was extracted with CH_2Cl_2 (3 × 20 mL), and the organic extracts were dried over $MgSO_4$, filtered off and evaporated under reduced pressure. The resulting isonitrile was used without any purification and dissolved in 600 µL tBuOH:$CHCl_3$ (3:2). Formic acid (8 µL, 0.198 mmol), formaldehyde (30% in H_2O, 40 µL, 0.198 mmol) and *N*-allylmethylamine (20 µL, 0.198 mmol) were added. The reaction mixture was stirred for 16 h and the solvent was removed under reduced pressure to yield a crude residue, which was purified by column chromatography (CH_2Cl_2) to afford **NIRBD-62** (14 mg, 31% yield).

^{1}H NMR (500 MHz, MeOD) δ 7.92 – 7.83 (m, 2H), 7.61 (d, *J* = 8.7 Hz, 2H), 7.60 – 7.52 (m, 2H), 7.43 – 7.38 (m, 2H), 7.40 – 7.33 (m, 2H), 7.01 (d, *J* = 8.8 Hz, 2H), 6.83 – 6.77 (m, 2H), 6.13 – 5.97 (m, 1H), 5.62 (d, *J* = 17.9 Hz, 1H), 5.36 (t, *J* = 4.6 Hz, 2H), 4.00 (s, 3H), 3.81 – 3.75 (m, 4H), 2.90 (s, 3H), 2.27 – 2.18 (m, 4H), 2.19 – 2.15 (m, 3H), 1.55 (s, 3H), 1.31 (s, 3H).

^{13}C NMR (126 MHz, MeOD) δ = 160.8, 152.8, 141.7, 138.7, 138.5, 137.7, 135.9, 134.0, 132.9, 129.4, 129.3, 128.5, 120.0, 117.3, 116.4, 115.3, 114.1, 109.6, 54.4, 53.4, 48.2, 48.0, 40.7, 29.2, 13.6.

HRMS (m/z): (M+H$^+$) calcd. for $C_{41}H_{42}BF_2N_4O_3$: 687.3240; found 687.3268.

Synthesis of **NIRBD-62a**

To a solution of 2,2'-((((1E,1'E)-(5,5-difluoro-10-(4-formamidophenyl)-1,9-dimethyl-5H-4l4,5l4-dipyrrolo[1,2-c:2',1'-f][1,3,2]diazaborinine-3,7-diyl)bis(ethene-2,1-diyl))bis(4,1-phenylene))bis(oxy))diacetamide (30 mg, 0.044 mmol) in 6 mL $CHCl_3$ under inert atmosphere, 53 µL (0.308 mmol) DIPEA were added. The resulting mixture was cooled down at 0˚C and $POCl_3$ (10 µL, 0.110 mmol) was added dropwise. The reaction was stirred in the cold for 3 h. Then, 10 mL of 2 M $NaHCO_3$ were added, and the mixture was stirred for 5 min. The aqueous layer was extracted with CH_2Cl_2 (3 × 20 mL), and the organic extracts were dried over $MgSO_4$, filtered and evaporated under reduced pressure. The resulting isonitrile was used without any purification and dissolved in 600 µL tBuOH:$CHCl_3$ (3:2). Formic acid (5 µL, 0.264 mmol), formaldehyde (30% in H_2O, 27 µL, 0.132 mmol) and *N*-allylmethylamine (13 µL, 0.132 mmol) were added. The reaction mixture was stirred for 16 h and the solvent was removed under reduced pressure to yield a crude residue, which was purified by column chromatography (CH_2Cl_2:MeOH, 9:1) to afford **NIRBD-62a** (6 mg, 18% yield).

^{1}H NMR (500 MHz, MeOD) δ 9.89 (s, 1H), 8.24 (s, 1H), 7.95 – 7.88 (m, 2H), 7.88 – 7.82 (m, 2H), 7.72 – 7.58 (m, 3H), 7.47 – 7.32 (m, 2H), 7.24 – 7.06 (m, 2H), 6.96 (m, 2H), 6.81 (d, *J* = 3.1 Hz, 2H), 6.07 – 5.95 (m, 1H), 5.35 – 5.18 (m, 3H), 4.64 (s, 2H), 4.58 (s, 3H), 3.95 (d, *J* = 10.5 Hz, 2H), 3.26 (s, 4H), 2.43 (s, 2H), 1.56 (s, 3H), 1.31 (s, 3H), 1.24 (s, 3H).

^{13}C NMR (126 MHz, MeOD) δ = 191.4, 172.3, 171.7, 170.4, 162.8, 158.6, 157.6, 155.5, 152.9, 146.5, 146.5, 139.0, 137.3, 135.7, 135.3, 134.6, 133.2, 131.6, 130.8,

130.5, 129.8, 129.6, 128. 9, 128.6, 120.2, 117.7, 117.5, 115.4, 115.0, 114.8, 114.6, 66.6, 66.5, 66.5, 60.4, 60.1, 53.1, 53.0, 41.8, 37.2, 29.7, 29.3, 13.6.

HRMS (m/z): ($M+H^+$) calcd. for $C_{43}H_{44}BF_2N_6O_5$: 773.3429; found 773.3427.

<u>Synthesis of **NIRBD-62b**</u>

To a solution of *N*-(4-(3,7-bis((*E*)-4-(2-(diethylamino)ethoxy)styryl)-5,5-difluoro-1,9-dimethyl-5H-4l4,5l4-dipyrrolo[1,2-c:2',1'-*f*][1,3,2]diazaborinin-10-yl)phenyl) formamide (50 mg, 0.065 mmol) in 6 mL $CHCl_3$ under inert atmosphere, 82 µL (0.455 mmol) DIPEA was added. The resulting mixture was cooled down at 0˚C and $POCl_3$ (15 µL, 0.195 mmol) was added dropwise. The reaction was stirred in the cold for 3 h. Then, 10 mL of 2 M $NaHCO_3$ were added, and the mixture was stirred for 5 min. The aqueous layer was extracted with CH_2Cl_2 (3 × 20 mL), and the organic extracts were dried over $MgSO_4$, filtered and evaporated under reduced pressure. The resulting isonitrile was used without any purification and dissolved in 600 µL tBuOH:$CHCl_3$ (3:2). Formic acid (7 µL, 0.39 mmol), formaldehyde (30% in H_2O, 39 µL, 0.195 mmol) and *N*-allylmethylamine (19 µL, 0.195 mmol) were added. The reaction mixture was stirred for 16 h and the solvent was removed under reduced pressure to yield a crude residue, which was purified by column chromatography (CH_2Cl_2:MeOH, 9:1) to afford **NIRBD-62b** (11 mg, 20% yield).

^{1}H NMR (500 MHz, MeOD) δ 8.55 (s, 1H), 7.87 (d, *J* = 8.3 Hz, 2H), 7.64 (d, *J* = 8.6 Hz, 4H), 7.58 (d, *J* = 16.4 Hz, 4H), 7.43 – 7.31 (m, 6H), 7.07 (d, *J* = 8.6 Hz, 4H), 6.81 (s, 2H), 6.07 – 5.93 (m, 1H), 5.38 – 5.20 (m, 2H), 4.32 (t, *J* = 5.1 Hz, 4H), 3.27 (s, 2H), 3.23 (d, *J* = 6.6 Hz, 2H), 3.08 (d, *J* = 6.9 Hz, 8H), 2.43 (s, 3H), 1.57 (s, 6H), 1.28 (t, *J* = 7.2 Hz, 12H).

^{13}C NMR (126 MHz, MeOD) δ 170.4, 168.6, 159.1, 152.6, 142.1, 139.0, 138.3, 135.6, 134.6, 133.1, 130.2, 129.0, 128.6, 120.2, 117.7, 117.4, 114.7, 63.7, 60.4, 60.1, 51.0, 41.8, 13.6, 8.7.

HRMS (m/z): (M+H$^+$) calcd. for $C_{51}H_{64}BF_2N_6O_3$: 857.5096; found 857.5040.

<u>Synthesis of **NIRBD-62c**</u>

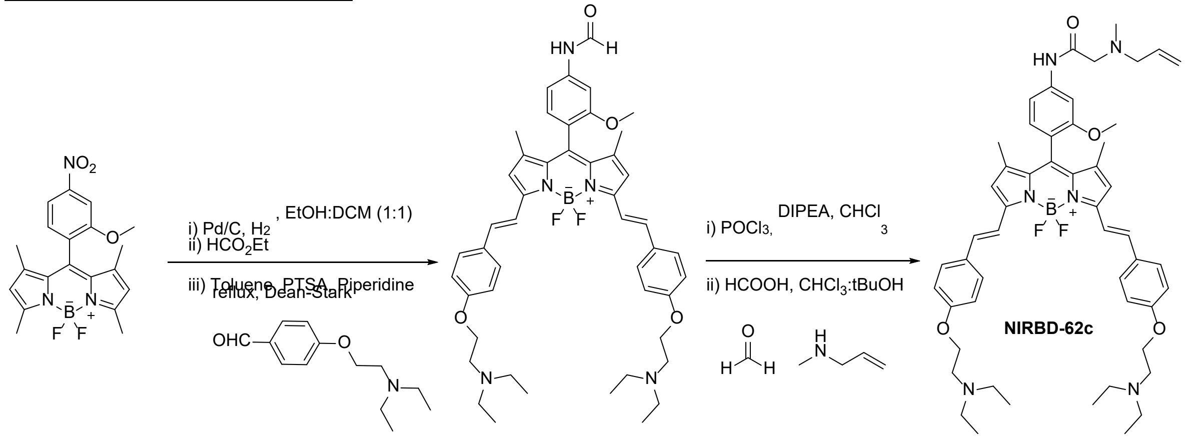

To a solution of 5,5-difluoro-10-(2-methoxy-4-nitrophenyl)-1,3,7,9-tetramethyl-5H-4l4,5l4-dipyrrolo[1,2-c:2',1'-f][1,3,2]diazaborinine (90 mg, 0.226 mmol) in 15 mL of degassed EtOH:CH_2Cl_2 (1:1), was added a suspension of Pd/C (10% mol) in EtOH under inert atmosphere. The resulting mixture was stirred at r.t. under H_2 (1 atm) for 10 h. When the reaction was completed, inorganic solids were removed by filtration through Celite® and washed with several portions of CH_2Cl_2. The solvent was removed under reduced pressure and the resulting amine, without any purification, was dissolved in ethyl formate (8 mL) and heated in a sealed tube at 80 ˚C for 4 days. When the reaction was completed, the solvent was removed under reduced pressure. The crude reaction mixture was dissolved in CH_2Cl_2 (15 mL) and washed with 1 M solution of NH_4Cl (3 × 7 mL). The organic extracts were dried over $MgSO_4$, filtered off and evaporated under reduced pressure. The residue was purified by

flash chromatography (hexane:EtOAc 1:1) to obtain 75 mg of the formamide derivative (84% yield). Next, in a 50 mL round bottom flask with a Dean-Stark N-(4-(3,7-bis((E)-4-(2-(diethylamino)ethoxy)styryl)-5,5-difluoro-1,9-dimethyl-5H-4l4,5l4-dipyrrolo[1,2-c:2',1'-f][1,3,2]diazaborinin-10-yl)-3-methoxyphenyl)formamide (50 mg, 0.126 mmol), 4-[2-(dimethylamino)ethoxy]-benzaldehyde (111 mg, 0.504 mmol), *p*-toluenesulfonic acid (10 mg, 0.06 mmol), toluene (20 mL) and piperidine (0.6 mL) were added. After heating to reflux, the reaction was monitored by TLC and the stopped when starting material disappeared completely. Then, the solvent was removed under reduced pressure and the residue was purified by column chromatography (CH_2Cl_2/MeOH 9:1) to afford the formamide derivative (53% yield). Then, to a solution of the N-(4-(3,7-bis((E)-4-(2-(diethylamino)ethoxy)styryl)-5,5-difluoro-1,9-dimethyl-5H-4l4,5l4-dipyrrolo[1,2-c:2',1'-f][1,3,2]diazaborinin-10-yl)-3-methoxyphenyl)formamide (54 mg, 0.067 mmol) in 6 mL $CHCl_3$ under inert atmosphere, 86 µL (0.470 mmol) DIPEA was added. The resulting mixture was cooled down at 0˚C and $POCl_3$ (16 µL, 0.201 mmol) was added dropwise. The reaction was stirred in the cold for 3 h. Then, 10 mL of 2 M $NaHCO_3$ were added, and the mixture was stirred for 5 min. The aqueous layer was extracted with CH_2Cl_2 (3 × 20 mL), and the organic extracts were dried over $MgSO_4$, filtered and evaporated under reduced pressure. The resulting isonitrile was used without any purification and dissolved in 600 µL tBuOH:$CHCl_3$ (3:2). Formic acid (8 µL, 0.402 mmol), formaldehyde (30% in H_2O, 41 µL, 0.201 mmol) and *N*-allylmethylamine (20 µL, 0.201 mmol) were added. The reaction mixture was stirred for 16 h and the solvent was removed under reduced pressure to yield a crude residue, which was

purified by column chromatography (CH_2Cl_2/MeOH 9:1) to afford **NIRBD-62c** (11 mg, 19% yield).

^{1}H NMR (500 MHz, $CDCl_3$) δ 8.53 (s, 1H), 7.74 (d, *J* = 1.8 Hz, 2H), 7.64 (d, *J* = 16.3 Hz, 2H), 7.59 (d, *J* = 8.7 Hz, 4H), 7.21 (d, *J* = 16.3 Hz, 2H), 7.13 (d, *J* = 8.1 Hz, 2H), 7.05 (dd, *J* = 8.1, 1.9 Hz, 2H), 6.94 (d, *J* = 8.8 Hz, 4H), 6.62 (s, 2H), 5.98 – 5.87 (m, 1H), 5.35 – 5.24 (m, 2H), 4.29 (t, *J* = 5.6 Hz, 4H), 3.83 (s, 3H), 3.26 – 3.08 (m, 2H), 2.94 (m, 8H), 2.45 (s, 3H), 2.29 (s, 3H), 1.58 (s, 6H), 1.25 (t, *J* = 7.2 Hz, 12H).

^{13}C NMR (126 MHz, $CDCl_3$) δ 169.4, 167.8, 158.9, 157.4, 152.3, 141.5, 140.2, 135.1, 134.3, 133.6, 131.1, 130.2, 129.0, 119.5, 118.9, 117.7, 117.2, 114.8, 111.4, 102.5, 65.0, 60.9, 57.2, 55.7, 51.0, 47.4, 43.3, 14.3, 10.4.

HRMS (m/z): ($M+H^+$) calcd. for $C_{52}H_{66}BF_2N_6O_4$: 887.5201; found 887.5224.

NMR spectra

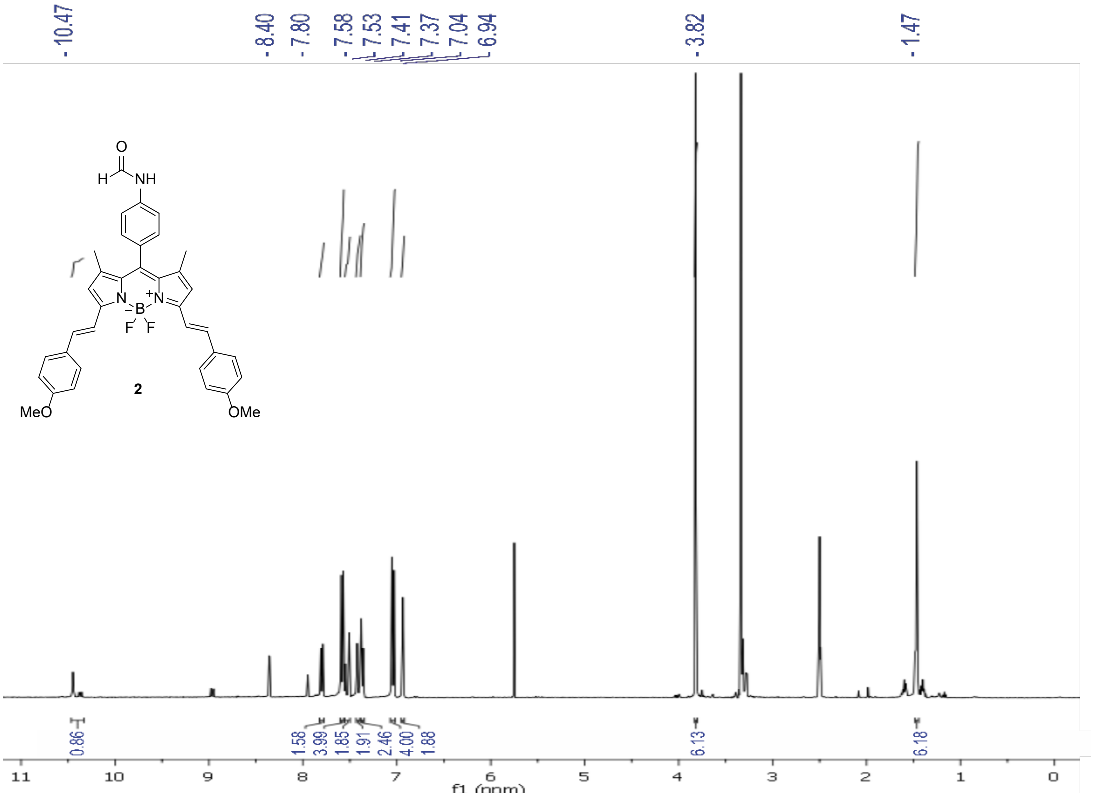

10.47
8.40
7.80
7.58
7.53
7.41
7.37
7.04
6.94
3.82
1.47
O
H
NH
N
B
N
F
F
2
MeO
OMe
0.86
1.58
3.99
1.85
1.91
2.46
4.00
1.88
6.13
6.18
11
10
9
8
7
6
5
4
3
2
1
0

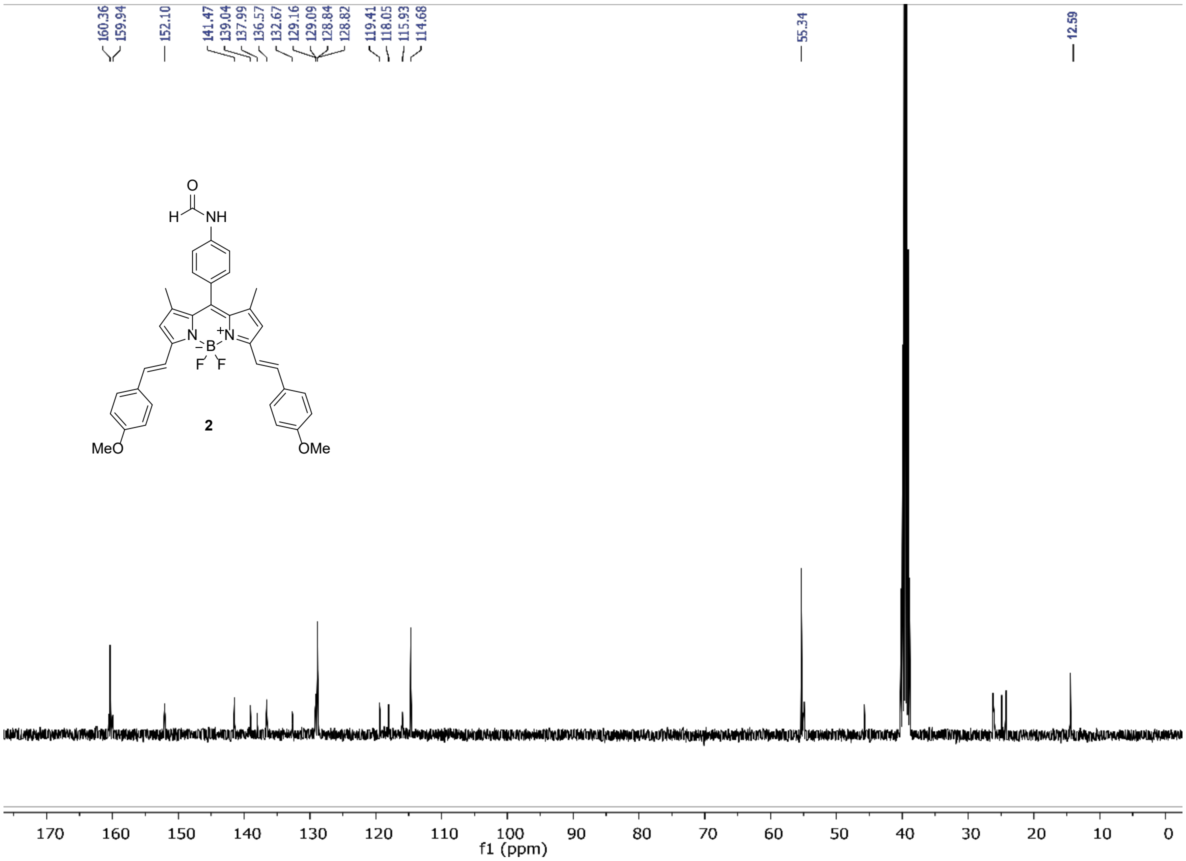

160.36
159.94
152.10
141.47
139.04
137.99
136.57
132.67
129.16
129.09
128.84
128.82
119.41
118.05
115.93
114.68
55.34
12.59
O
H
NH
N
B
N
F
F
2
MeO
OMe
170
160
150
140
130
120
110
100
90
80
70
60
50
40
30
20
10
0
f1 (ppm)

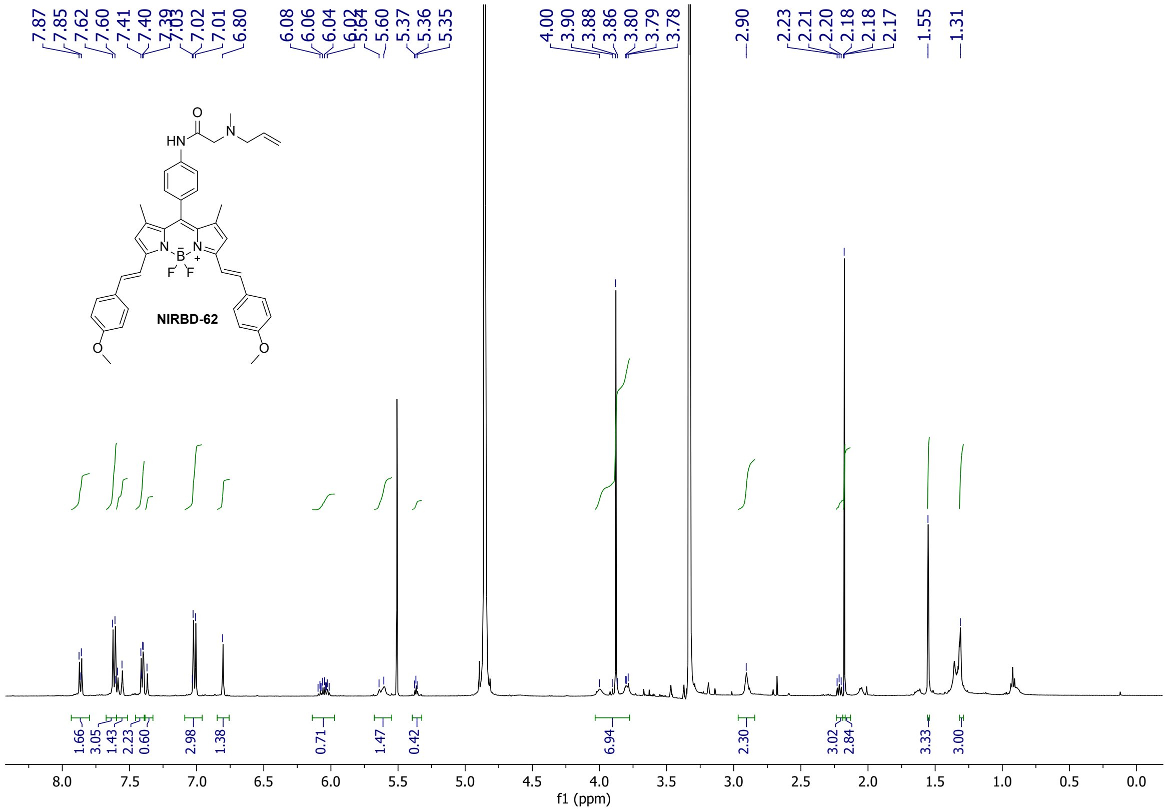
7.87 7.85 7.62 7.60 7.41 7.40 7.39 7.03 7.02 7.01 6.80 6.08 6.06 6.04 6.02 5.64 5.60 5.37 5.36 5.35 4.00 3.90 3.88 3.86 3.80 3.79 3.78 2.90 2.23 2.21 2.20 2.18 2.18 2.17 1.55 1.31
NIRBD-62
1.66 3.05 1.43 2.23 0.60 2.98 1.38 0.71 1.47 0.42 6.94 2.30 3.02 2.84 3.33 3.00
f1 (ppm)

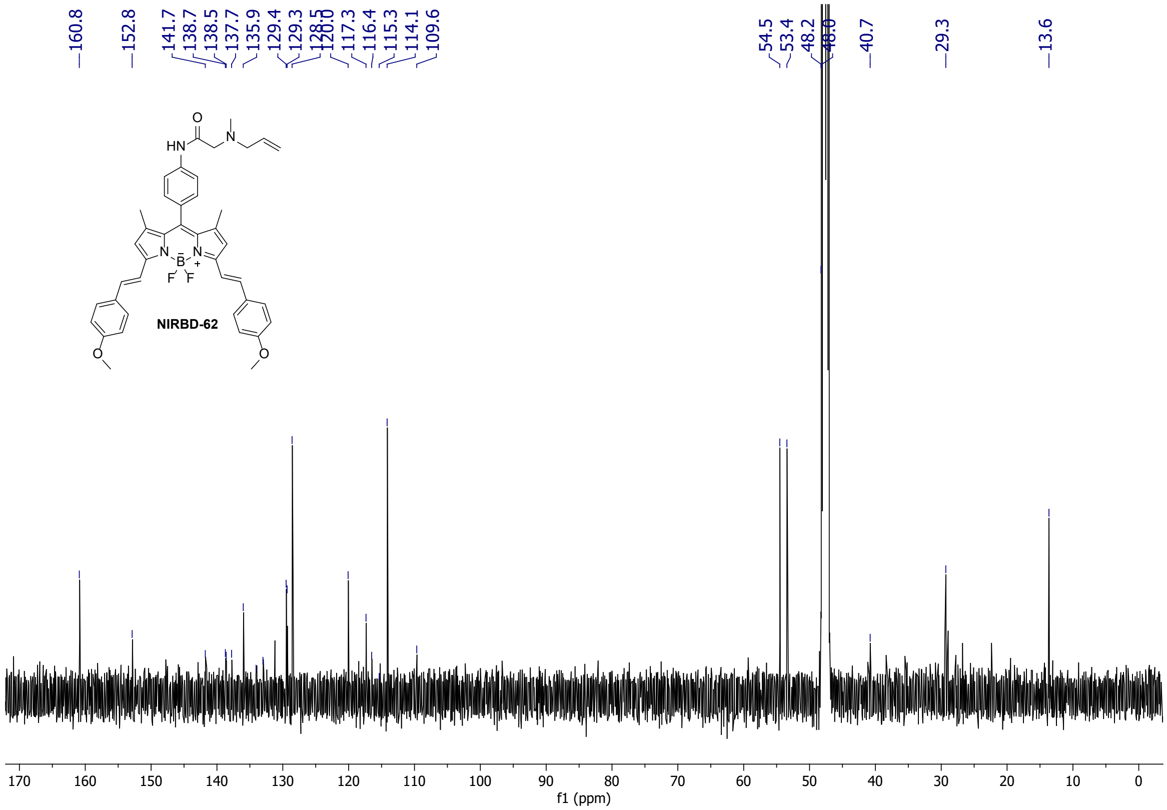
160.8 152.8 141.7 138.7 138.5 137.7 135.9 129.4 129.3 128.5 120.0 117.3 116.4 115.3 114.1 109.6 54.5 53.4 48.2 48.0 40.7 29.3 13.6
NIRBD-62
f1 (ppm)

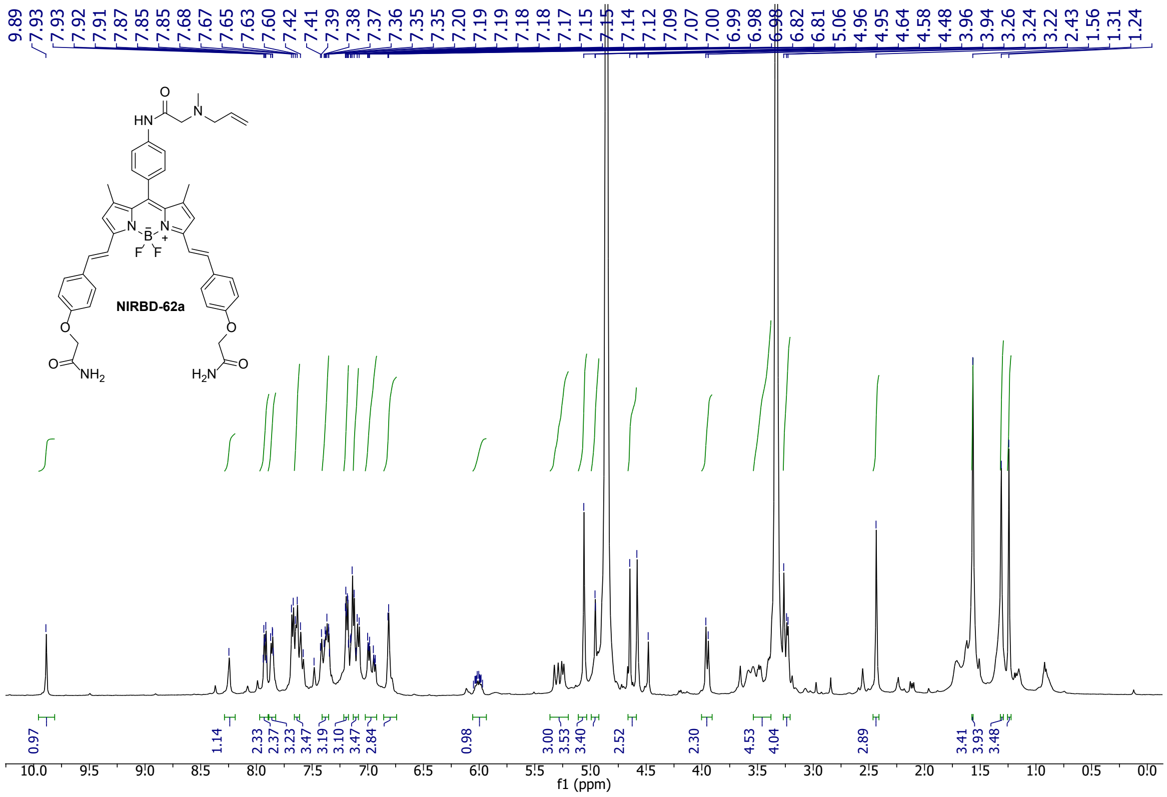
9.89
7.93
7.93
7.92
7.91
7.87
7.85
7.85
7.68
7.67
7.65
7.63
7.60
7.42
7.41
7.39
7.38
7.37
7.36
7.35
7.35
7.20
7.19
7.19
7.18
7.18
7.17
7.15
7.15
7.14
7.12
7.09
7.07
7.00
6.99
6.98
6.98
6.82
6.81
5.06
4.96
4.95
4.64
4.58
4.48
3.96
3.94
3.26
3.24
3.22
2.43
1.56
1.31
1.24
HN
O
N
N
B
N
F F
NIRBD-62a
O
O
O NH2
H2N O
0.97
1.14
2.33
2.37
3.23
3.47
3.19
3.10
3.47
2.84
0.98
3.00
3.53
3.40
2.52
2.30
4.53
4.04
2.89
3.41
3.93
3.48
10.0 9.5 9.0 8.5 8.0 7.5 7.0 6.5 6.0 5.5 5.0 4.5 4.0 3.5 3.0 2.5 2.0 1.5 1.0 0.5 0.0
f1 (ppm)

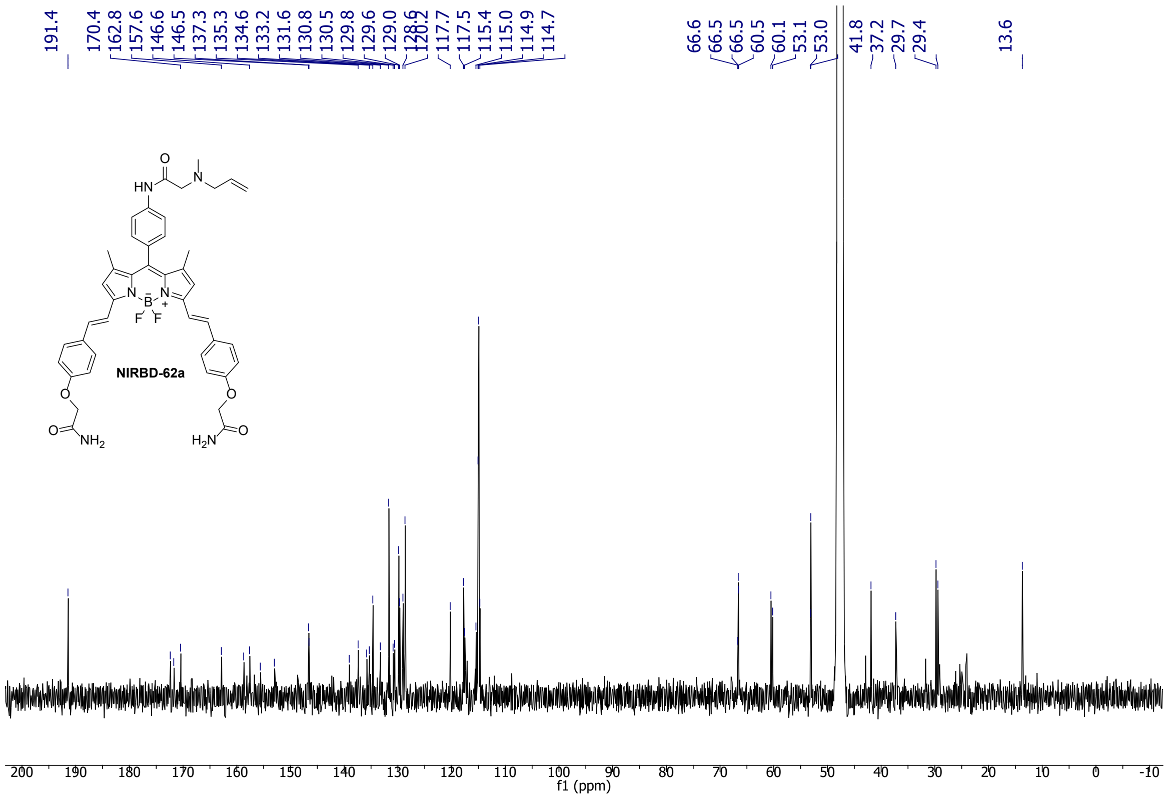
191.4
170.4
162.8
157.6
146.6
146.5
137.3
135.3
134.6
133.2
131.6
130.8
130.5
129.8
129.6
129.0
128.6
128.2
117.7
117.5
115.4
115.0
114.9
114.7
66.6
66.5
66.5
60.5
60.1
53.1
53.0
41.8
37.2
29.7
29.4
13.6
HN
O
N
N
B
N
F F
NIRBD-62a
O
O
O NH2
H2N O
200 190 180 170 160 150 140 130 120 110 100 90 80 70 60 50 40 30 20 10 0 -10
f1 (ppm)

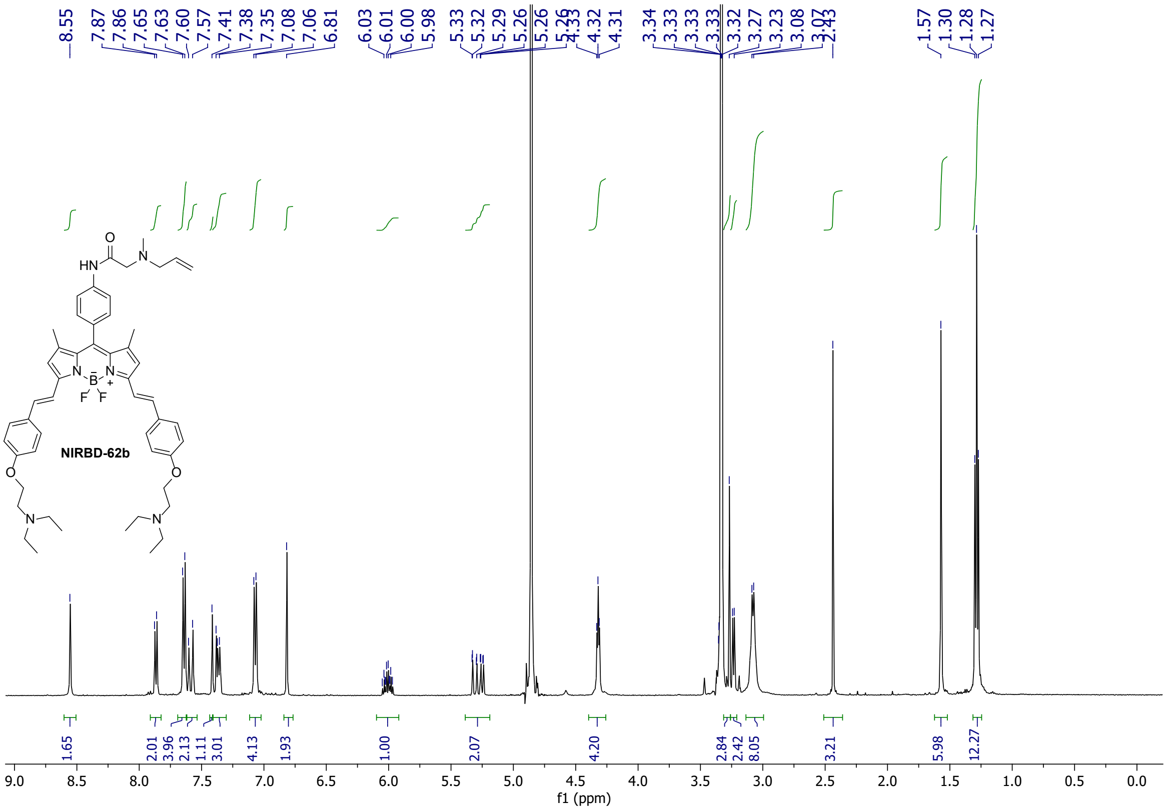
8.55
7.87 7.86 7.65 7.63 7.60 7.57 7.41 7.38 7.35 7.08 7.06 6.81
6.03 6.01 6.00 5.98
5.33 5.32 5.29 5.26 5.26 5.26 4.33 4.32 4.31
3.34 3.33 3.33 3.33 3.32 3.27 3.23 3.08 3.07 2.43
1.57 1.30 1.28 1.27
1.65 2.01 3.96 2.13 1.11 3.01 4.13 1.93 1.00 2.07 4.20 2.84 2.42 8.05 3.21 5.98 12.27
NIRBD-62b
f1 (ppm)

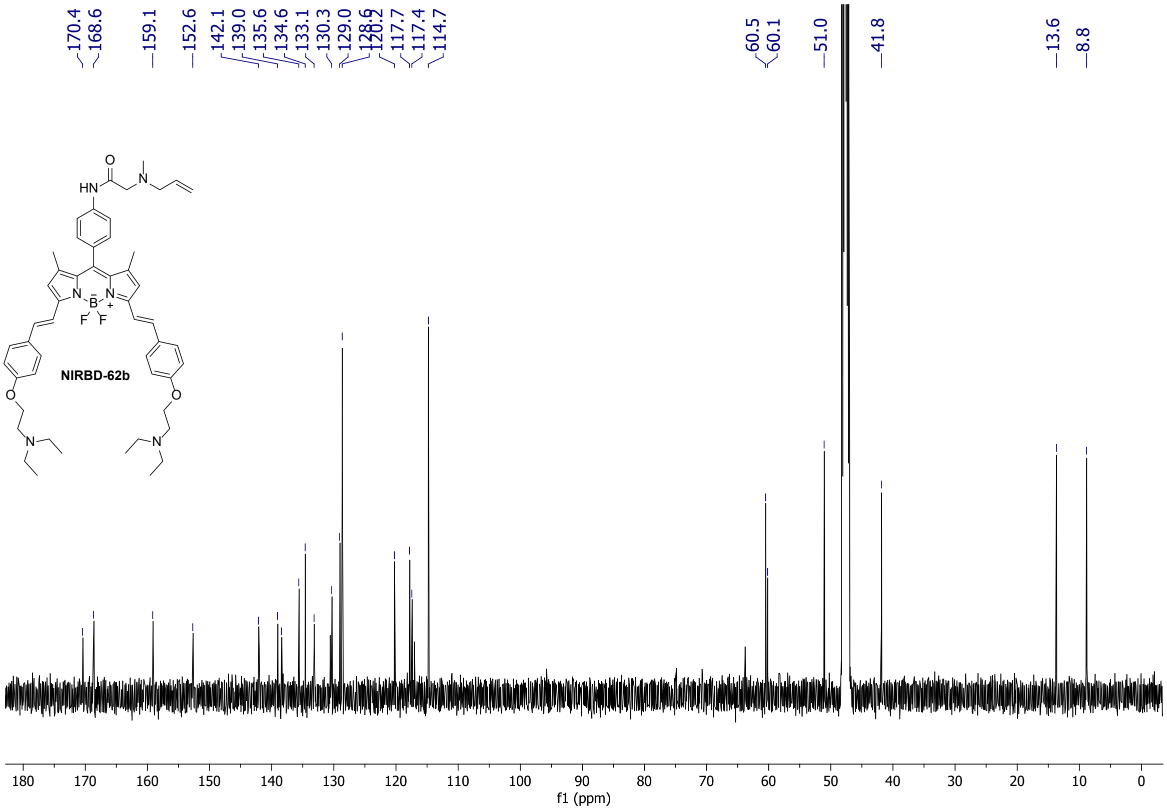
170.4 168.6 159.1 152.6 142.1 139.0 135.6 134.6 133.1 130.3 129.0 128.5 120.2 117.7 117.4 114.7
60.5 60.1 51.0 41.8 13.6 8.8
NIRBD-62b
f1 (ppm)

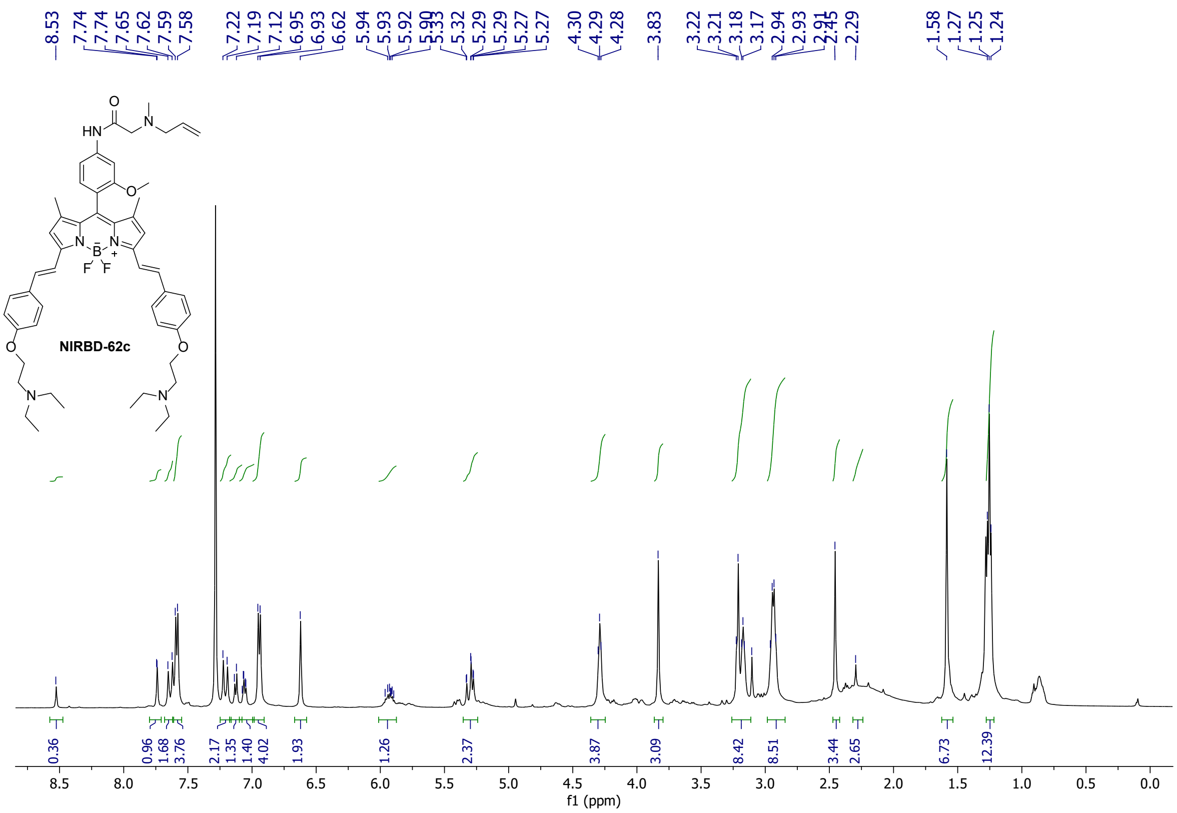
8.53 7.74 7.74 7.65 7.62 7.59 7.58 7.22 7.19 7.12 6.95 6.93 6.62 5.94 5.93 5.92 5.90 5.33 5.32 5.29 5.29 5.27 5.27 4.30 4.29 4.28 3.83 3.22 3.21 3.18 3.17 2.94 2.93 2.91 2.45 2.29 1.58 1.27 1.25 1.24
HN
O
N
O
N
B
N
F F
O
O
N
N
NIRBD-62c
0.36 0.96 1.68 3.76 2.17 1.35 1.40 4.02 1.93 1.26 2.37 3.87 3.09 8.42 8.51 3.44 2.65 6.73 12.39
8.5 8.0 7.5 7.0 6.5 6.0 5.5 5.0 4.5 4.0 3.5 3.0 2.5 2.0 1.5 1.0 0.5 0.0
f1 (ppm)

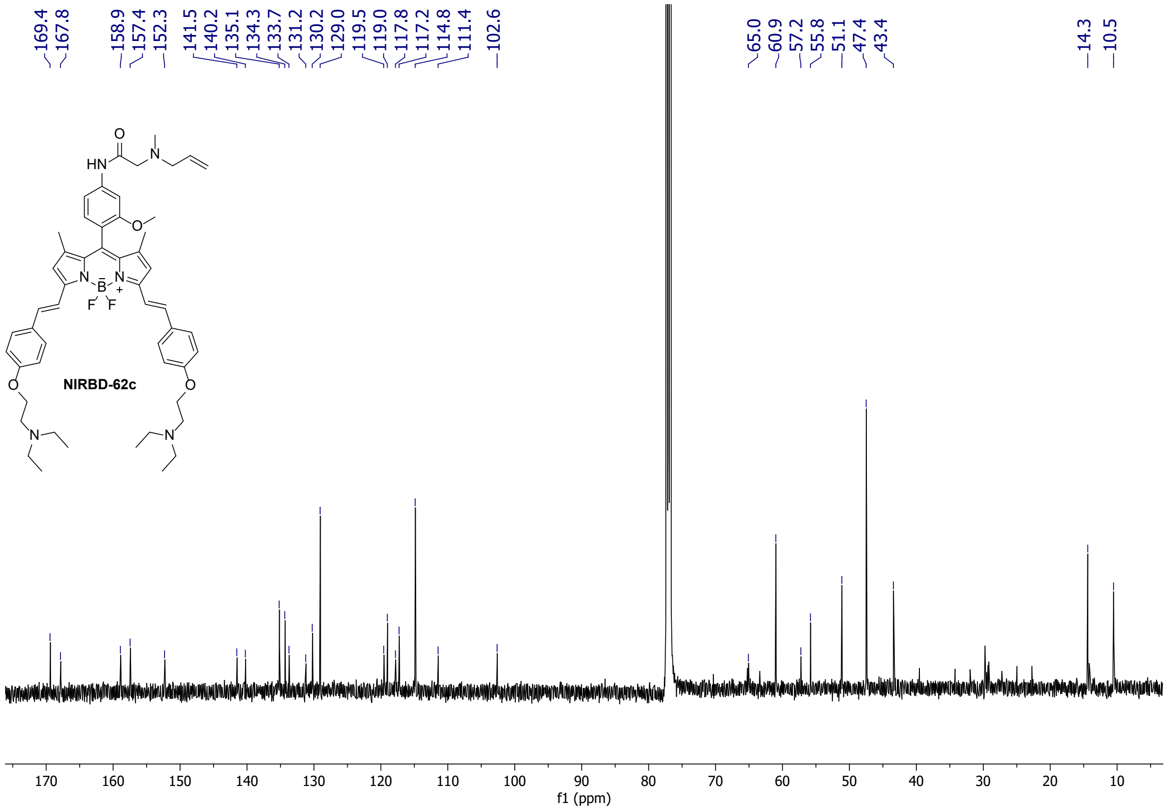
169.4 167.8 158.9 157.4 152.3 141.5 140.2 135.1 134.3 133.7 131.2 130.2 129.0 119.5 119.0 117.8 117.2 114.8 111.4 102.6 65.0 60.9 57.2 55.8 51.1 47.4 43.4 14.3 10.5
HN
O
N
O
N
B
N
F F
O
O
N
N
NIRBD-62c
170 160 150 140 130 120 110 100 90 80 70 60 50 40 30 20 10
f1 (ppm)

Spectroscopy and imaging protocols

Spectroscopy instrumentation. A double-beam spectrophotometer UV-Vis (Perkin Elmer, LAMBDA 650) was employed for absorption spectra acquisition. For steady-state fluorescence measurements we employed a Jasco FP-8300 spectrofluorometer. Excitation and emission spectra of compounds were determined in solvents of different polarity. The spectra were recorded between 300 and 750 nm with excitation set at the appropriate excitation wavelength. Slit widths were set to 2.5 nm for excitation and 2.5 nm for emission. All spectra were corrected for background fluorescence by subtracting a blank scan of the solvent solution.

Fluorescence quantum yields. Fluorescence quantum yields (Φ) were determined using Rhodamine 101 in MeOH (Φ = 1.0) as the reference. A solution of the fluorophore was compared to the standard to confirmed that the variation in absorbance units was less than 0.1 at identical excitation wavelengths. The following equation was used to calculate the quantum yields:

$$\Phi = \frac{I_x \cdot A_r \cdot n_x^2}{I_r \cdot A_x \cdot n_r^2} \cdot \Phi_r$$

where *x* and *r* denote the sample and standard, respectively, *A* is the absorption at the excitation wavelength, *I* is the integrated fluorescence intensity, and *n* is the refractive index of the solvent.

Fluorescence lifetimes. Time-resolved fluorescence decays of solutions were determined on a PicoQuant FT200 fluorescence lifetime spectrometer based on single photon timing (SPT) using a 635-nm pulsed diode laser as excitation source (PicoQuant LDH-635) at a pulse repetition rate of 20 MHz.

Fluorescence decay histograms were collected at λ_{max}^{em}–10 nm, λ_{max}^{em}, and λ_{max}^{em}+10 nm with a time resolution of 36 ps/channel until 2×10^4 counts were collected in the peak channel. Fluorescence lifetimes were obtained from iterative deconvolution global fits to a biexponential decay function of the three fluorescence decay traces using the FluoFit software (PicoQuant). The standard errors were obtained from the diagonal elements of the covariance matrix available from the global fits and were between 5 and 30 ps. The instrument-response function (IRF) was collected using LUDOX scatterer.

Cell cultures. Bone-marrow derived macrophages (BMDMs) were isolated and derivatized as previously reported.[2] Mouse osteoblasts (MC3T3-E1) were grown in Dulbecco's modified Eagle's medium (DMEM) supplemented with 10% (v/v) fetal bovine serum (FBS), 2 mM glutamine plus 100 U mL^{-1} penicillin and 0.1 mg mL^{-1} streptomycin. Cell cultures were provided by the Cell Culture Facility of University of Granada and maintained in an incubator at 37 °C with 95 % humidity and 5 % CO_2.

Cell imaging. BMDMs were plated in 96-well plates and incubated without or with IFNγ (100 U mL^{-1}) overnight and with NIRBD compounds (250 nM) and Hoechst 33342 (1 µM) for 1 h at 37 ºC. Cells were washed with PBS and imaged under ImageXpress™ XLS. Fluorescence images (4 sites/well) were acquired under a 20× objective using DAPI and Cy5 excitation filters and data was analyzed with MetaXpress software using the Custom Module Editor.

For dual-color FLIM experiments, cells were seeded onto circular coverslips (diameter of 25 mm) in 6-well plates at a density of 2.3×10^5 cells per well. Dual-color FLIM was carried out using a MicroTime 200 fluorescence-lifetime microscope

(PicoQuant) equipped with a pulsed, 635-nm excitation laser (LDH-635, PicoQuant), working at 20 MHz. The collected emission was spatially filtered through a 75-μm pinhole and separated into two detection channels (single-photon avalanche diodes, SPCM-AQR 14, Perkin Elmer) with bandpass filters of 685/70 and 750/40, respectively. Photon time-tagging was performed on a TimeHarp 200 module (PicoQuant). For experiments at low temperature, once the cell cultures reached the desired confluence on the chambered slides, they were incubated for 10 min at 4°C; then compound **NIRBD-62c** (100 nM) was added and further incubated for 10 min at 4°C. Finally, cells were washed off with PBS to remove any excess of dye and taken for fluorescence imaging at room temperature. Control experiments were performed, following the same incubation and washing steps but at 37 °C.

FLIM analysis was performed in SymphoTime 64 (PicoQuant) and home-coded ImageJ routines.[3] FLIM images were fitted to a biexponential decay model, after a 4×4 pixels spatial binning was applied, fixing the shorter lifetime at 1.5 ns to account for cellular autofluorescence. FLIM was reconstructed with the long lifetime, that belongs exclusively to the dye. Ratiometric images were obtained by dividing the I_{685} image by the I_{750} image.

Confocal and STED images of **NIRBD-62c** in osteoblasts and co-localization studies were performed on an Abberior Expert Line equipped with a pulsed excitation laser of 640 nm, working at a repetition rate of 20 MHz for confocal and FLIM images. For STED imaging, a pulsed, toroidal, depletion laser of 775 nm was used. Fluorescence emission was recorded on a silicon avalanche photodiode using a 685/70 bandpass

filter. For STED-FLIM imaging, single photon time-tagging to reconstruct the pixel fluorescence decay was performed on a HydraHarp 400 module (PicoQuant).

Supplementary Figures

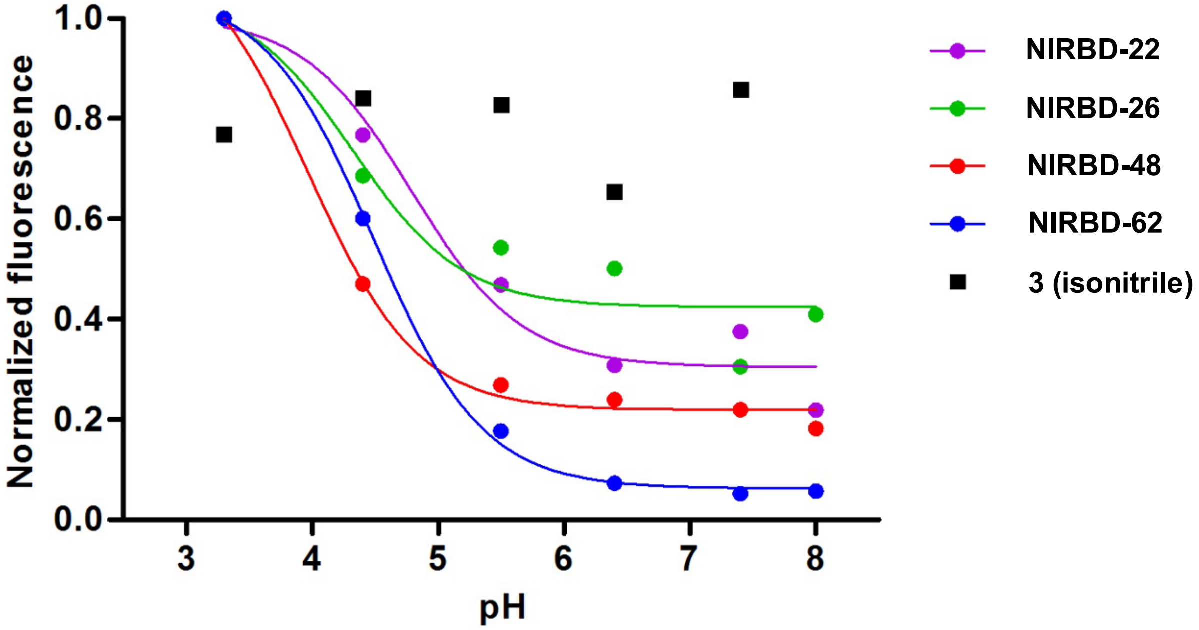

Figure S1. pH sensitivity of selected NIRBD fluorophores. NIRBD compounds and the pH-insensitive isonitrile **3** (10 μM) were dissolved in phosphate buffers of different pH. Values represented as averages from two independent experiments.

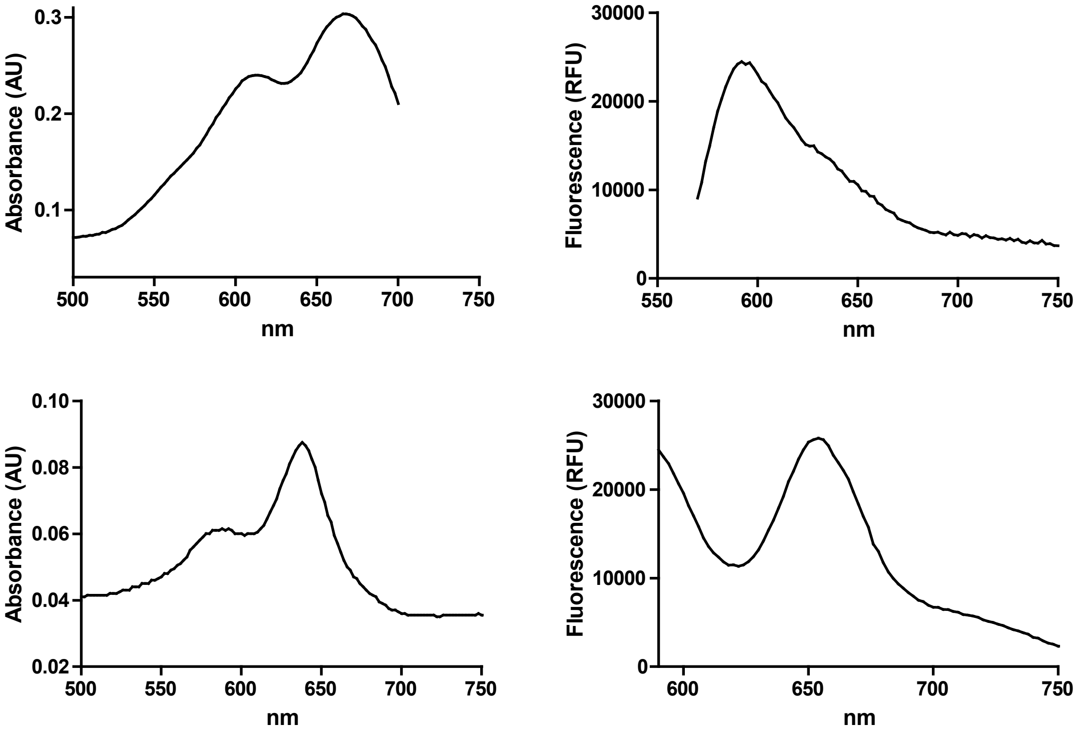

Figure S2. Absorbance and emission spectra of NIRBD fluorophores. NIRBD-62 (top) and NIRBD-62b (bottom) were dissolved in PBS (10 μM) and spectra are represented as averages from two independent experiments.

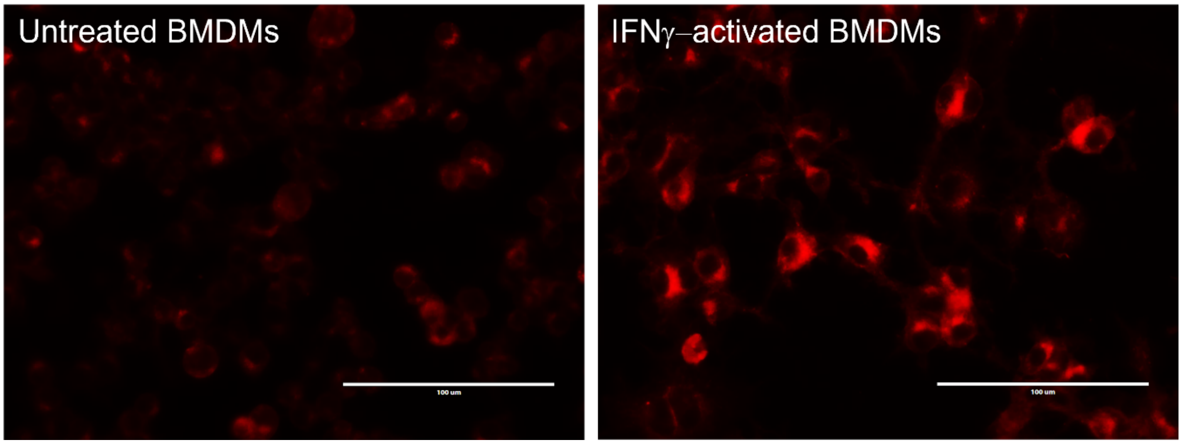

Figure S3. Fluorescence microscopy images of BMDMs. Murine BMDMs were isolated as described in the experimental section and stimulated or not with IFNγ (100 U mL^{-1}). Both cultures were incubated with compound **NIRBD-62b** (250 nM) and imaged under an epifluorescence microscopy (Cy5 filter). Scale bar: 100 μm.

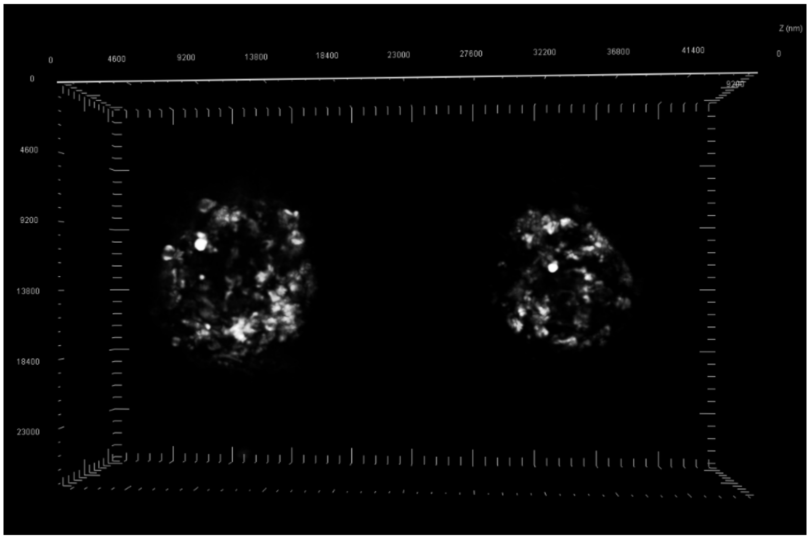

Figure S4. Z-stack fluorescence microscopy images of murine macrophages. RAW264.7 cells were stimulated with IFNγ (100 U mL^{-1}), incubated with compound **NIRBD-62b** (250 nM) and imaged under an AiryScan confocal microscopy (Cy5 filter) to confirm intracellular localization.

solvent	ϕ
cyclohexane	0.68 ± 0.09
1,4-dioxane	0.99 ± 0.05
chloroform	0.9 ± 0.3
chlorobenzene	0.42 ± 0.02
tetrahydrofuran	0.85 ± 0.12
dichloromethane	0.99 ± 0.05
acetone	0.59 ± 0.02
2–propanol	0.55 ± 0.02
ethanol	0.46 ± 0.02
methanol	0.39 ± 0.01
water	0.25 ± 0.02

Figure S5. Fluorescence quantum yield values of compound **NIRBD-62c** in different solvents. Quantum yields were measured at excitation wavelengths of 578 and 585 nm using the relative method and Rhodamine 101 in methanol as reference.[4]

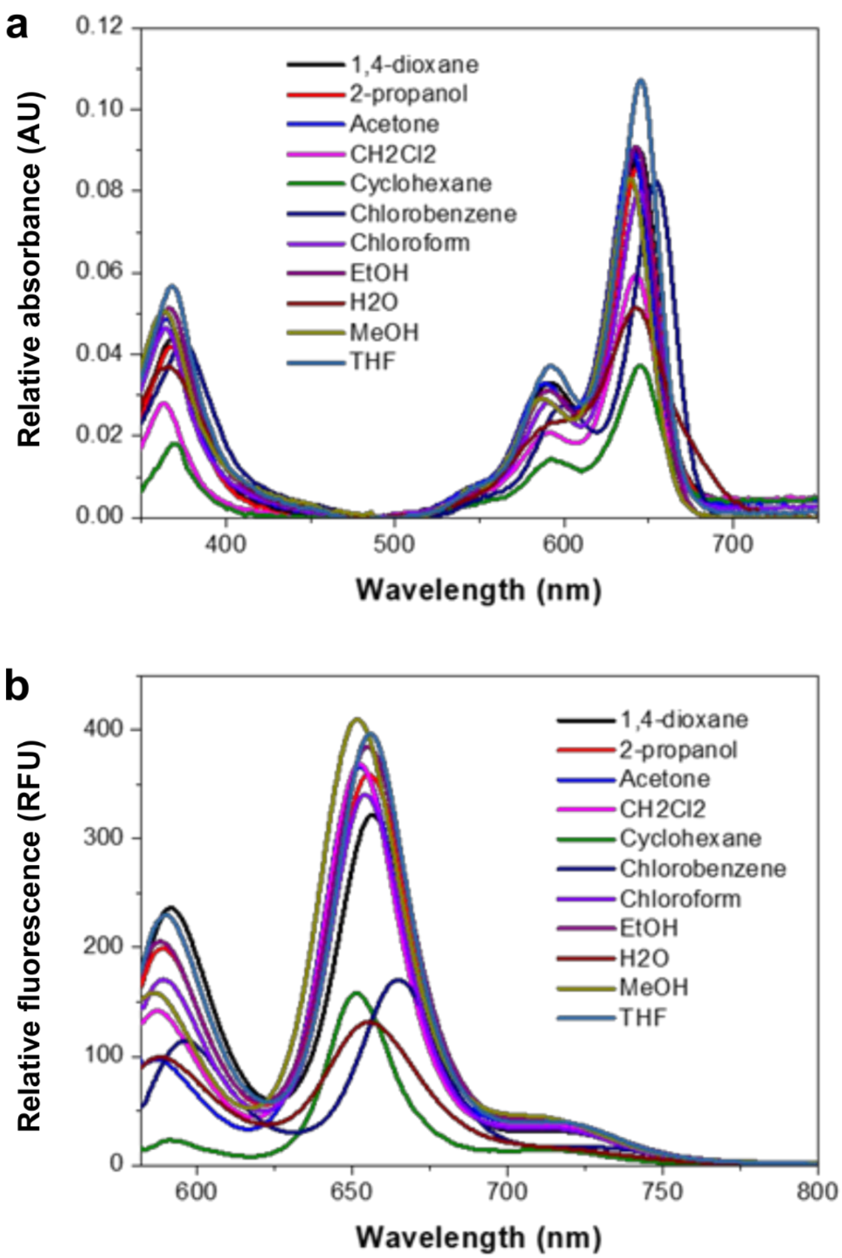

Figure S6. Absorbance (a) and fluorescence emission (b) spectra of compound **NIRBD-62c** in different organic solvents. Emission spectra were acquired after excitation at 578 nm.

Photophysical parameter	a (SA)	b (SB)	c (SP)	d (SdP)	y_0	r^2
$\frac{\bar{\upsilon}_{abs}}{cm^{-1}}$	-180±82	-439±146	**-2878±685**	-179±146	17922±619	0.93
$\frac{\bar{\upsilon}_{em}}{cm^{-1}}$	-224±97	-394±173	**-2238±812**	-2±173	17049±735	0.86

Figure S7. Coefficients of the multilinear correlation analysis of the absorption and emission transition energies (in terms of wavenumbers, $\bar{\upsilon}_{abs}$ and $\bar{\upsilon}_{em}$) of the compound **NIRBD-62c** in the solvent scales of polarity (SdP), polarizability (SP), acidity (SA), and basicity (SB), according to equation below. This methodology, proposed by Catalán,[5] isolates the solvent's characteristics that are the most defining for the dye's spectroscopic behavior. For both absorption and emission of **NIRBD-62c**, the coefficient with the highest weight (highlighted in bold) was the solvent's polarizability.

$$\bar{\upsilon}_i = y_0 + aSA + bSB + cSP + dSdP$$

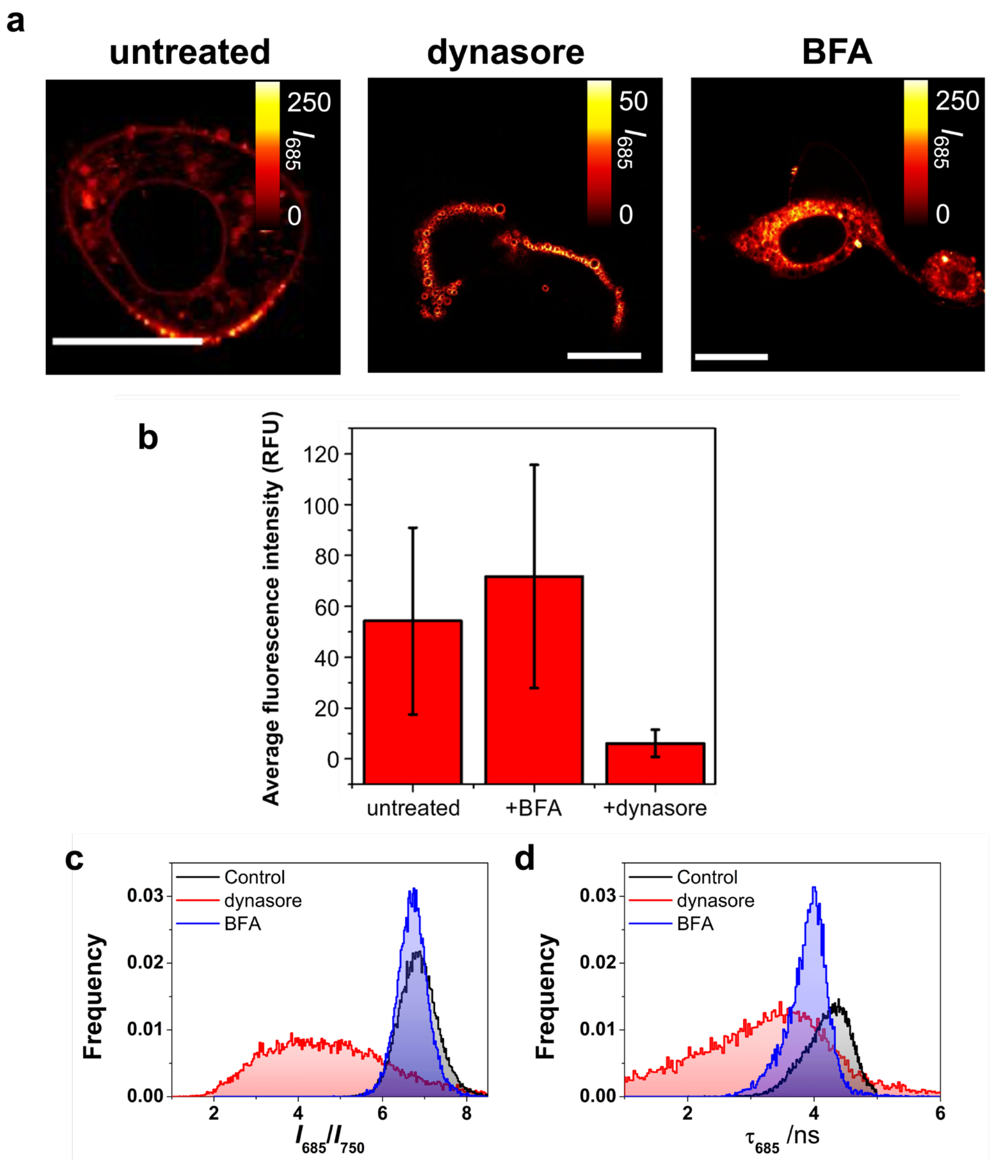

Figure S8. a) Representative pseudocolored fluorescence microscopy images (emission 685 nm) of osteoblasts (all incubated with **NIRBD-62c**, 100 nM) that were untreated, treated with dynasore (80 μM) or BFA (5 μM) and b) average fluorescence intensities and histograms presented as means±SD. Scale bar: 20 μm. c, d) Frequency histograms of the I_{685}/I_{750} ratio (c) and fluorescence lifetimes (d) obtained from multiparametric fluorescence microscopy experiments. Histograms contain the contribution of 10 different images for each condition.

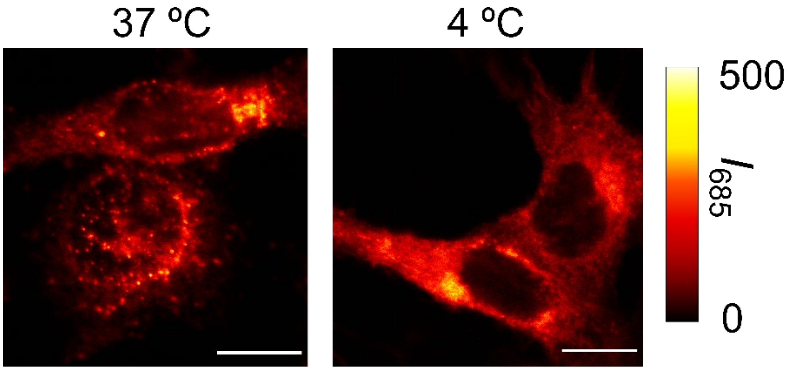

Figure S9. Representative fluorescence microscopy images of MC3T3-E1 osteoblasts upon incubation with compound **NIRBD-62c** (100 nM) at 37 ºC or 4 ºC. STED images were acquired using three pulses of excitation + depletion pulses of 640 nm excitation laser. Emission was collected using a 685/70 nm bandpass filter. Scale bar: 10 μm.

Supplementary Movies

Time-lapse FLIM of live osteoblasts after incubation with **NIRBD-62c** represented in pseudocolored movies showing variable fluorescence lifetimes in different vesicular compartments. Images were acquired every 10 s for a total of 10 min.

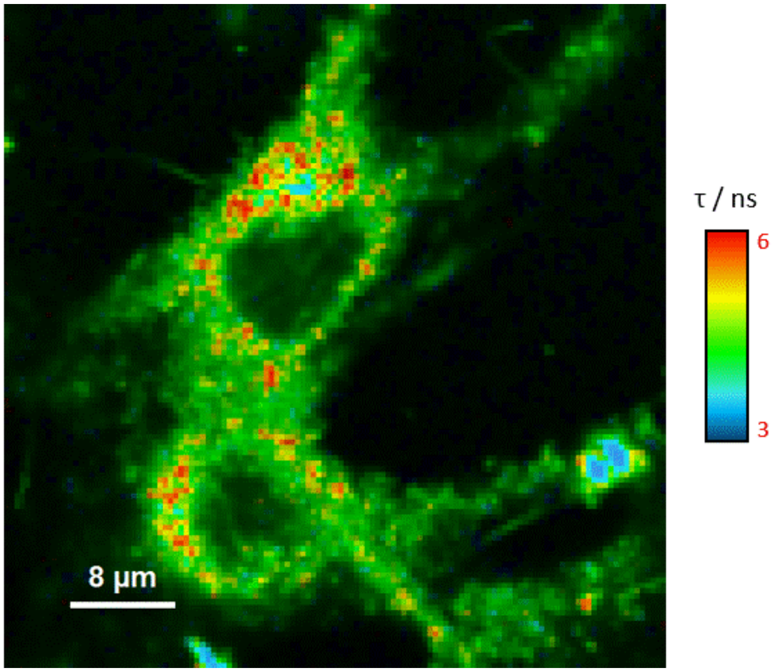

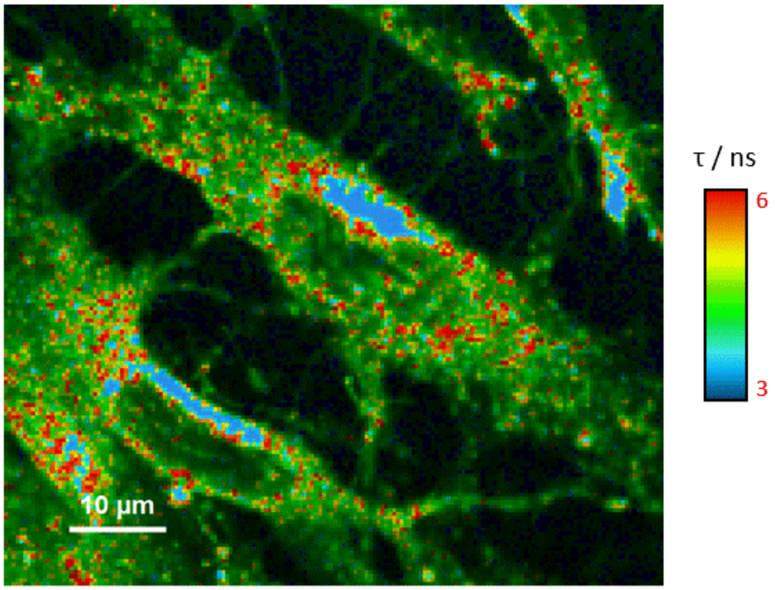

Supplementary References

[1] A. Vázquez-Romero et al. *J. Am. Chem. Soc.*, 2013, **135**, 16018-16021.

[2] Guc, E. et al. *Methods Enzymol.* 2020, **632**, 91-111.

[3] Schindelin, J. et al. *Nat. Methods* 2012, 9, 676–82.

[4] Brouwer, A. M. *Pure Appl. Chem.* 2011, **83**, 2213-2228.

[5] J. Catalán, *J. Phys. Chem. B*, 2009, **113**, 5951-5960.
